# EndoGene database: reported genetic variants for 5,926 Russian patients diagnosed with endocrine disorders

**DOI:** 10.3389/fendo.2025.1472754

**Published:** 2025-02-18

**Authors:** Anton A. Buzdin, Marianna A. Zolotovskaia, Sergey A. Roumiantsev, Aleksandra G. Emelyanova, Olga O. Golounina, Polina A. Pugacheva, Daniil V. Luppov, Anastasia V. Kuzminyh, Arseniya O. Alexeeva, Anna A. Emelianova, Alexey L. Novoselov, Alina Matrosova, Anastasia A. Slepukhina, Sergey V. Popov, Evgeniya V. Plaksina, Vasiliy M. Petrov, Anastasia R. Guselnikova, Anastasia D. Shagina, Maria V. Suntsova, Victoriya V. Zakharova, Zhanna E. Belaya, Maria V. Vorontsova, Galina A. Melnichenko, Natalia G. Mokrysheva, Vladimir P. Chekhonin, Ivan I. Dedov

**Affiliations:** ^1^ Laboratory of Bioinformatics, Endocrinology Research Center, Moscow, Russia; ^2^ Laboratory of Clinical and Genomic Bioinformatics, I.M. Sechenov First Moscow State Medical University, Moscow, Russia; ^3^ Moscow Center for Advanced Studies, Moscow, Russia; ^4^ Laboratory of Systems Biology, Shemyakin-Ovchinnikov Institute of Bioorganic Chemistry, Moscow, Russia; ^5^ Laboratory for Personalized Medicine, Institute of Chemical Biology and Fundamental Medicine, Novosibirsk, Russia; ^6^ Department of Internal Medicine, Lomonosov Moscow State University, Moscow, Russia

**Keywords:** genetic database, endocrine pathology, mutations, diabetes mellitus, Mendelian diseases, human genetic variants

## Abstract

**Introduction:**

Endocrine system disorders are a serious public health burden and can be caused by deleterious genetic variants in single genes or by the combined effects of multiple variants along with environmental and lifestyle factors.

**Methods:**

The EndoGene database presents the results of next-generation sequencing assays used to genetically profile 5,926 patients who were diagnosed with 450 endocrine and concomitant diseases and were examined and treated at the National Medical Research Center for Endocrinology between November 2017 and January 2024. Among them, 494, 1,785, 692, and 1,941 patients were profiled using four internally developed genetic panels including 220, 250, 376, and 382 genes, respectively, selected based on a literature analysis and clinical recommendations, and 1,245 patients were profiled by whole exome sequencing covering 31,969 genes.

**Results:**

2,711 genetic variants were reported as clinically relevant by medical geneticists and are presented here along with genomic, technical, and clinical annotations.

**Discussion:**

This publicly accessible database will be useful to those interested in genetics, epidemiology, population statistics, and a better understanding of the molecular basis of endocrine disorders.

## 1 Introduction

Endocrine diseases, including diabetes, thyroid dysfunction, and other hormonal imbalances, contribute significantly to the global burden of disease (1). These diseases not only affect public health but also lead to long-term disability and reduced quality of life for the affected individuals (1). The prevalence of these disorders is increasing, especially in the context of an aging population and the increasing incidence of metabolic disorders (2, 3).

These disorders can be caused by rare variants in a single gene (Mendelian or monogenic diseases), by the combined effects of multiple genetic variants, or by environmental and lifestyle factors (polygenic diseases such as type 2 diabetes mellitus or obesity). New techniques such as gene therapy offer hope when diseases cannot be effectively treated with traditional drugs. This is possible when the etiology of the inherited disease is known. Thus, a functional copy of a gene is introduced into the human body with the help of a gene therapy drug, slowing down the progression of the disease and, in some cases, even achieving significant improvement (4). In recent years, advancements in technology have facilitated the characterization of genomic diversity across a wide range of populations (5). Next-generation sequencing (NGS) and genome-wide association studies (GWASs) have been intensely used to study the genetic basis of endocrine diseases (6–9). However, the interpretation of identified variants using criteria widely recommended by the American College of Medical Genetics and the Association for Molecular Pathology (ACMG/AMP) (10) is challenging because detailed phenotypic information associated with specific variants is limited in most databases (11). To improve the accuracy of diagnosis, prognosis, and genetic counseling, the importance of variant databases in patients with specific diagnoses (12) is increasingly recognized. Such databases constitute systematically organized repositories of genetic variants, supplemented with clinical data (13). They facilitate communication between researchers, clinicians, and patients by allowing the sharing of information about genes, variants, and pathologic phenotypes (11).

Previous studies have created databases that include genetic variants associated with specific endocrinopathies. For example, the MEN2 RET database developed by Margraf et al. is a publicly accessible database that contains all *RET* sequence variants related to MEN2 syndromes as well as relevant clinical data (14). The “NGS and PPGL Study Group” also collected and classified variants in the *SDHB* gene, which is one of the major genes responsible for paraganglioma/pheochromocytoma predisposition (PPGL), leading to the creation of the *SDHB* variant database (15). In Argentina, a study of 170 patients with congenital hypopituitarism identified causative variants in both known and recently proposed candidate genes (9). In addition, a recent report presented a database containing comprehensive experimentally validated associations between endocrine diseases and long non-coding RNAs (16).

However, it is important to consider the potential role of population-specific variants in disease pathogenesis. Uncommon variants tend to be specific to certain populations (17). It has been observed that disease-causing variants often exhibit population specificity not only for rare but also for common diseases, which emphasizes the importance of considering pedigree in genetic studies and clinical diagnosis (18). The multinational population of the Russian Federation, comprising more than one hundred different ethnic groups, demonstrates genetic heterogeneity (19–21) and provides a unique but challenging opportunity to study the genetic basis of inherited pathogenic mutations and their contribution to disease etiology in different populations. A recent study presented a database on the frequency of genetic variants in Russia (22). In addition, several databases have been created for Russian patients with hereditary cancer syndromes (23, 24).

The aim of our study was to create the first representative database of genetic variants specifically targeting endocrine diseases in the Russian population. We collected information on pathogenic, likely pathogenic, and other genetic variants identified by panel NGS and/or whole exome sequencing (WES) in 5,926 patients with various endocrine pathologies. The database includes information on zygosity and pathogenicity classification according to ACMG/AMP recommendations and the presence of reported variants in previous scientific publications and in population frequency databases at the time of genetic interpretation. We also calculated gene mutation frequencies associated with each type of diagnosis.

In addition, we calculated the proportion of WES and smaller genetic panel analyses that resulted in the identification of variants for each type of endocrine diagnosis, allowing us to compare the performance of WES and panel target sequencing tests.

We believe that our database and the analysis of the statistics of reported genetic variants will contribute to a better understanding of the genetic basis of endocrine diseases, aid in the interpretation of mutations found in different populations, and suggest changes in the composition of diagnostic NGS panels to increase their informative power.

The ability to predict clinical outcomes based on genetic data may be improved by identifying pathogenic variants specific to certain populations (25). This study is the first to establish the frequency of pathogenic variants in Russian patients with endocrine diseases. To our knowledge, the presented database is also one of the world’s largest genetic experimental knowledge bases on endocrine pathology. It contributes to the growing body of knowledge on the genetic basis of these diseases and opens the way for more accurate and personalized diagnosis and treatment.

## 2 Methods

### 2.1 Participant characteristics

The sample includes 5,926 patients who were subjected to NGS DNA sequencing tests performed in the National Medical Research Center for Endocrinology (Moscow) from November 2017 to January 2024. The patients either suffered from endocrine pathology or had unfavorable hereditary history. In all cases, written informed consent to participate in this study was acquired from the patients or from their legal representatives. The consent procedure and the design of the study were approved by the ethics committee of the National Medical Research Center for Endocrinology, Moscow, Russia.

Inclusion criteria were the availability of diagnosis and record with sequencing results interpreted by clinical geneticists according to the ACMG/AMP guidelines (10). Patients were not specifically selected based on their clinical diagnoses. However, given the specialization of the Endocrinology Research Center, the testing cohort predominantly included individuals with endocrine or endocrine-related pathology, and their relatives were considered potential carriers of pathogenic genetic variants. A complete set of ICD10 diagnoses associated with individual patients and specific genetic variants is available in the database file (https://doi.org/10.5281/zenodo.10894526) and the patients can be filtered by ICD10 code for specific disease types.

Exclusion criteria were records with genetic variants that were not confirmed by two or more identifiers or were not classified according to ACMG guidelines (e.g., due to the need for additional examination of the patient).

### 2.2 Library preparation and sequencing

Genomic DNA was extracted using a NucleoMag Blood Kit (Macherey−Nagel), MagPure Blood Dna, Kit (Magen), MagPure Universal Dna Kit (Magen), or HiPure Universal Dna Kit (Magen). DNA concentrations were measured on Qubit 4 fluorimeter. Library preparation was performed using a KAPA HyperPlus Kit (Roche), VAHTS Universal Plus DNA Library Prep Kit for Illumina V2 (Vazyme), or Illumina DNA Prep with Enrichment reagents (Illumina). To allow sample multiplexing, indexed primers or adapters were used as follows: KAPA UDI Primer Mixes (Roche), VAHTS DNA Adapters for Illumina (Vazyme), and IDT for Illumina UD Indexes (IDT). For target enrichment, DNA libraries were hybridized with biotinylated DNA probes for 16 to 18 h and then captured by streptavidin beads. Hybridization and capture procedures were performed according to the KAPA HyperCap Workflow, VAHTS Target Capture Hybridization and Wash protocol, оr Illumina DNA Prep with Enrichment protocol with respective reagent kits. For whole exome enrichment, KAPA HyperExome Probes (Roche), a VAHTS Target Capture Core Exome Panel (Vazyme), or an IDT xGen Exome Hyb Panel (IDT) were used. Additionally, four custom probe panels were used for the enrichment of genes involved in endocrine disorders: Endo1, Endo2, Endome1, and Endome2 (Roche, designed in the National Medical Research Center for Endocrinology). Library quality was assessed using a 5200 Fragment Analyzer system (Agilent) with NGS Fragment Kits (1 to 6000bp). PE100 sequencing was performed on an Illumina NovaSeq 6000, NextSeq550, or MiSeq depending on the required number of reads per sample. The average mean exon coverage of x100 was obtained for both whole exome and target panel sequencing. Demultiplexing was performed using the Illumina Bcl2fastq2 program.

### 2.3 Data processing

**Figure 1:**
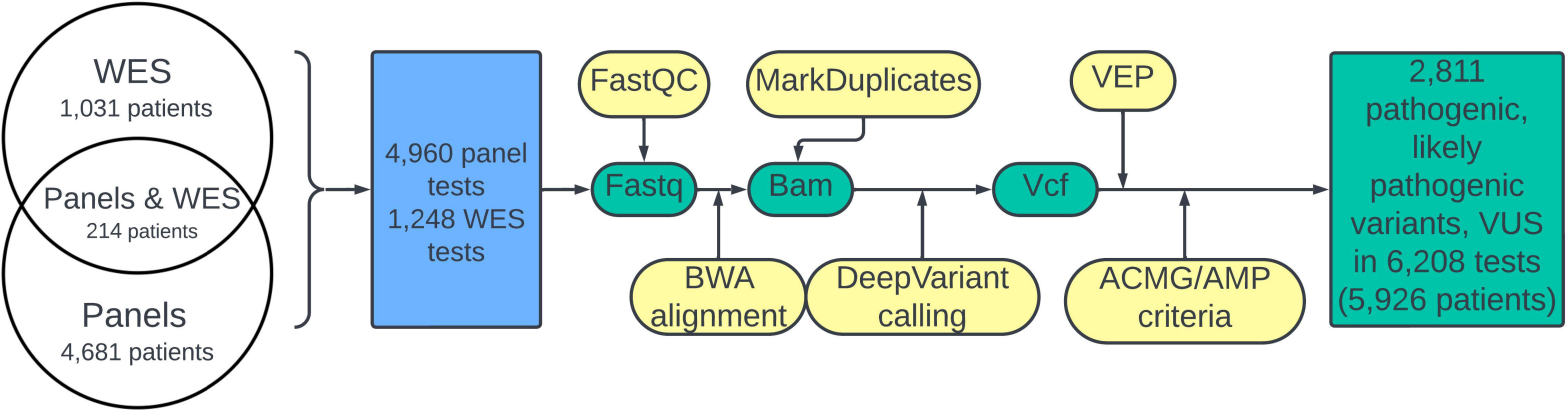
Flowchart of the study. The green color denotes molecular data; bioinformatic pipeline steps are shown in yellow. The blue block corresponds to performed NGS tests.

The design of the study is schematized in **Figure 1**. A quality check of the fastq files was done using FastP (26). The reads were aligned to the human genome assembly GRCh38 using BWA-mem (27). Coordinates of target regions correspond to the enrichment used. BAM coverage was calculated against the BED file using mosdepth. Samtools software was used for BAM file indexing. Duplicate marking was performed using MarkDuplicates software. We used DeepVariant for variant calling (28). All variants with an allele frequency in the experimental read for a particular biosample of less 0.01 were removed from further analysis. VCF annotation was performed using the VEP (29) tool. Variant interpretation was performed in accordance with the ACMG/AMP guidelines considering information about clinical features including phenotype and family segregation, VEP annotation, which characterized its potential impact on protein function (variant type, scores from *in silico* predictors CADD, PolyPhen, BayesDel, MutPred, MetaRNN, SpliceAI, and LoF), and data from population and clinical databases (gnomAD, ClinVar, and HGMD public). A complete list of VEP annotation fields is available in **Supplementary File 1**. In addition, information from variant-related scientific articles found in the PubMed database was used to annotate the fields.

### 2.4 Designs of target panels for NGS

**Figure 2:**
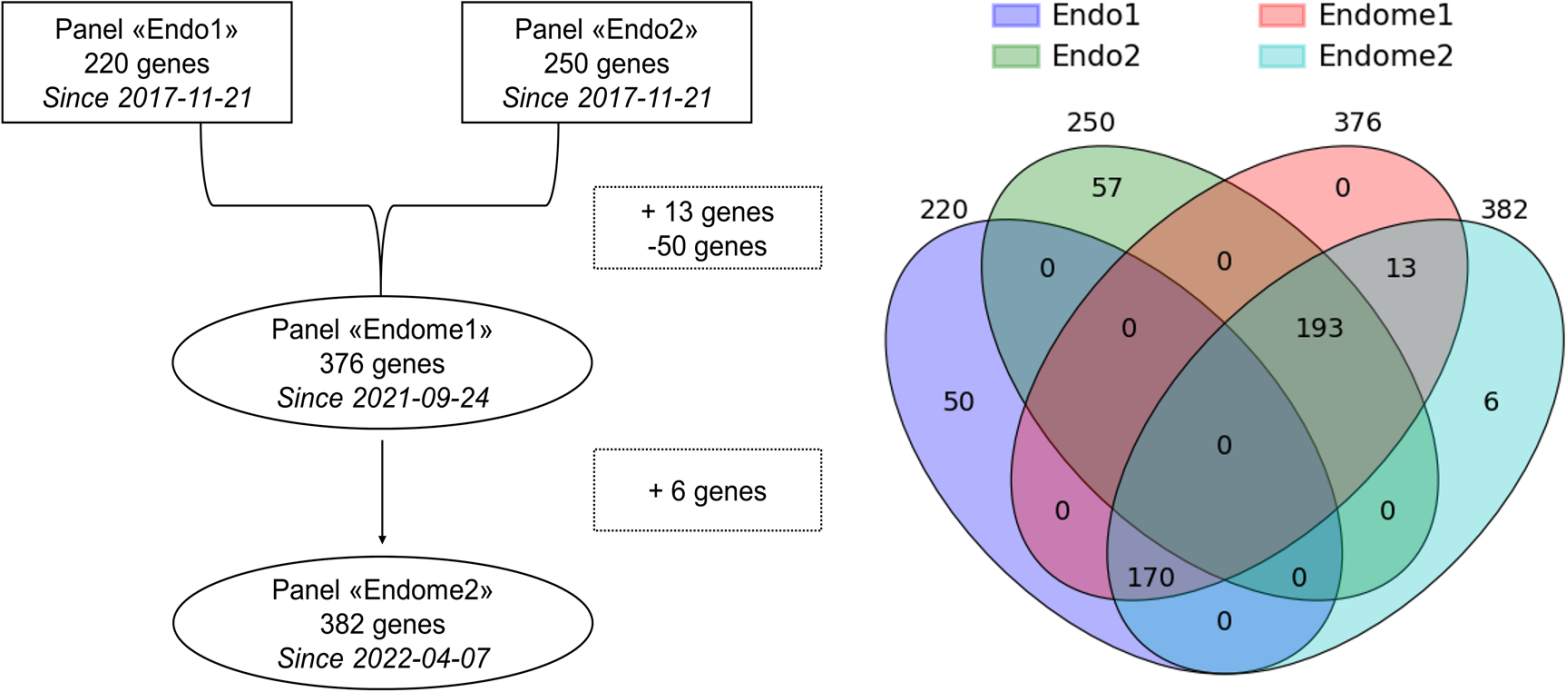
Relationship between the NGS panels used in this study. Intersections reflect the gene composition of panels under comparison.

The targeted NGS panels were developed at the National Medical Research Center for Endocrinology to cover genes known to be associated with endocrine pathologies. Initially, at the beginning of the project, two separate NGS panels, called Endo1 and Endo2, were developed. Later, they were combined with some modifications into one comprehensive Endome1 panel, which was further expanded to the Endome2 panel (**Figure 2**). The composition of the genes in the used NGS panels is given in **Supplementary File 2**.

### 2.5 Text analysis

Interpreted genetic variants were available as text records in electronic medical cards. Genetic coordinates, type of mutation, gene name, zygosity, and novelty of variant at the moment of interpretation were parsed with R v4.3.1 (30) and checked manually. Diagnoses of patients were automatically downloaded from the “ICD10 code” fields in the electronic medical cards. If the “ICD10 code” field was empty, the diagnoses were extracted manually from another field in the electronic medical cards or available hard medical documents.

### 2.6 Patient diagnoses

Every patient case was assigned an ICD10 code of diagnosis according to the 10th revision of the International Statistical Classification of Diseases and Related Health Problems, a medical classification list created by the World Health Organization. The code of the last available clinical diagnosis before the sequencing was used. If information about concomitant diagnoses was available, we also included the ICD10 codes for them. If the patient had no documented evidence about their pathology or any medical consultation at the moment on sequencing, ICD10 code Z01.8 was assigned.

### 2.7 Database format

We created a single comma-separated file with the following columns: “Patient ID”, “Age”, “Gender”, “ICD-10 code of the disease”, “Sequencing type”, “Panel design (if available)”, “Variant reported”, “Gene”, “Zygosity”, and “Described in the literature”. If at least one variant was reported for a patient, each row corresponded to one variant. If the patient had no reported variants, one row corresponded to one patient and the fields for the reported variant were empty. All the ICD-10 codes for the patients are listed in each row with a semicolon as a separator.

### 2.8 Technical validation

#### 2.8.1 Quality control of sequencing data

A data quality check was conducted on an Illumina SAV. A quality check of fastq files was conducted using FastP. All Illumina DNA short reads had a Phred score greater than 35 corresponding to a base accuracy greater than 99.9%.

#### 2.8.2 Quality control of archive data

Metadata from the laboratory information system, such as WES or NGS panel version, were manually compared with the information from the text descriptions of the sequencing results. All the information obtained through text parsing was manually verified.

To prevent any operator mistakes, we validated the parsed variant description. We considered the variant valid if one of the following conditions was met:

1. The variant was written in both genomic and transcriptomic coordinates. We ensured that both types of coordinates described the same variant.
2. The variant had a dbSNP ID. We checked if the dbSNP variant indeed matched the variant parsed from the geneticist’s report.
3. A *vcf* file was available and included the variant parsed from the geneticist’s report.

To match genomic and cDNA coordinates, dbSNP ID, and vcf records, we used the Mutalyzer (31) and VariantValidator (32) tools.

Additionally, we used protein coordinates, HGMD ID, or PubMed ID (variant description from a scientific article) in manual mode to confirm the parsed variant.

In this study, we did not include any results obtained using a bioinformatic pipeline other than that outlined in **Figure 1**. Genetic variants with incomplete information (genome assembly, genomic coordinates, or ACMG/AMP classification) were filtered out and not included in the database. In this study, we did not consider genetic variants without final classification with only partially met ACMG/AMP criteria.

#### 2.8.3 Control of clinical data

Interpretation of sequencing results for the individual patients was performed by clinical geneticists considering patient phenotype, medical documentation, and familial history when available. The correspondence of the patient diagnoses with the pathology group and with the results of NGS analysis was determined by the clinical endocrinologist.

## 3 Results and discussion

### 3.1 Overview of data records

We identified a total of 6,208 medical records with sequencing results for 5,926 patients. In total, 1,248 WES tests were performed for 1,245 patients, and 4,960 gene panel NGS tests were performed for 4895 patients. For 214 patients, both panel and WES tests were done. Some patients were tested several times due to technical or clinical reasons. Only genetic variants classified as “pathogenic,” “likely pathogenic,” or “of uncertain significance” were taken into consideration. Hereafter, they will be referred to as “reported variants”. The complete database file is available at the following link: https://doi.org/10.5281/zenodo.10894526.

**Figure 3:**
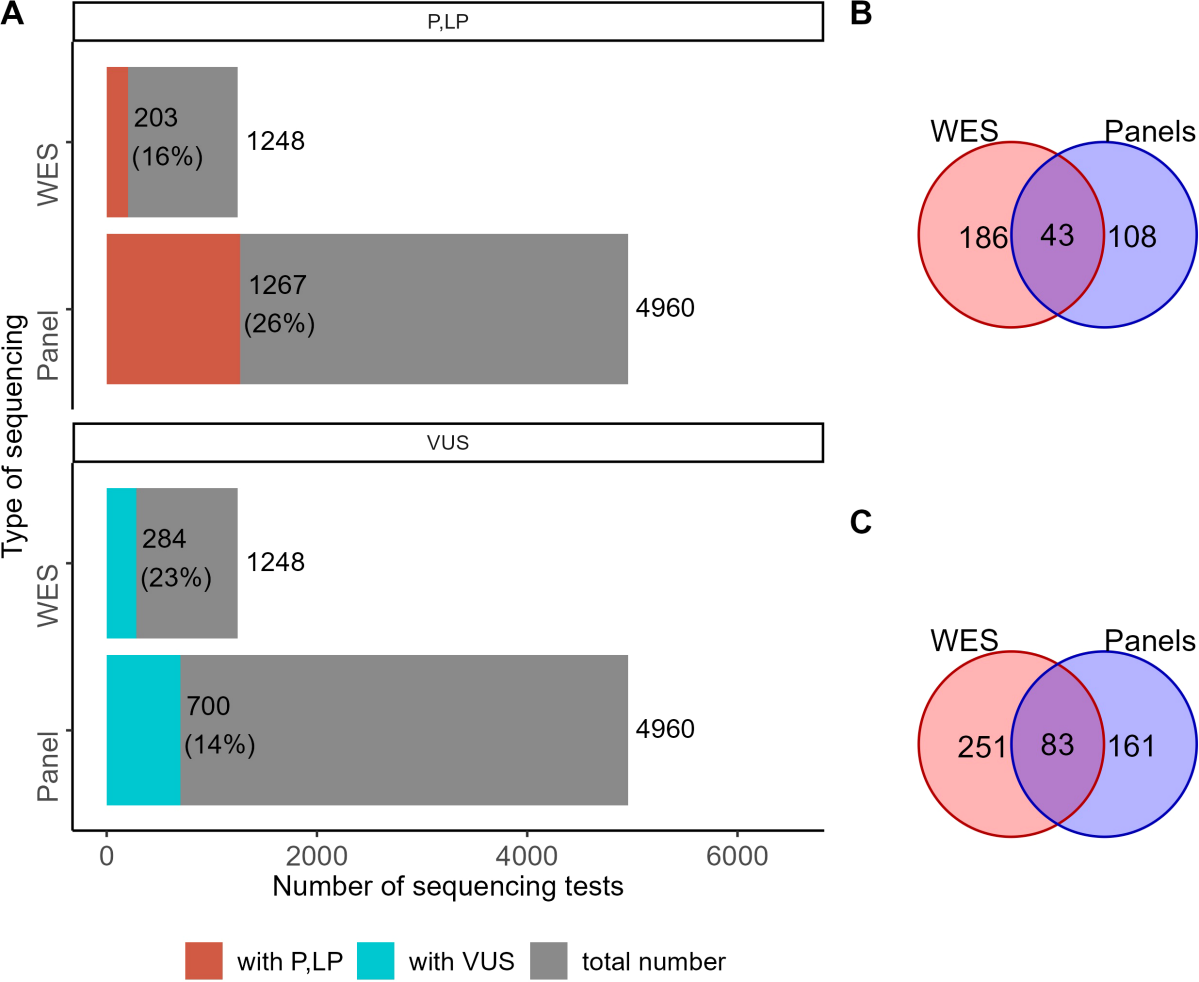
The proportion of NGS tests with reported genetic variants. **(A)** Number and percentage share of genetic tests with reported variants classified as “pathogenic” (P), “likely pathogenic” (LP), or “uncertain significance” (VUS) among the results of WES and panel NGS. **(B)** The number of genes hosting genetic variants classified as pathogenic or likely pathogenic in the results of WES and panel NGS. **(C)** The number of genes hosting genetic variants classified as VUS in the results of WES and panel NGS.

Relevant genetic variants were reported by clinical geneticists in 1,882 cases out of 4,960 NGS panel sequencing tests. Among them, 1,267 reports contained genetic variants classified as “pathogenic” and “likely pathogenic”, and 700 were “variants of uncertain significance” (**Figure 3**). For WES tests, relevant genetic variants were reported for 448 out of 1,248 tests, including pathogenic and likely pathogenic variants in 203 cases and variants of uncertain significance in 284 cases (**Figure 3**). In some patient cases (267 for panel NGS and 129 for WES), more than one variant was annotated and reported. Interestingly, the percentage share of the cases with reported genetic variants was very similar for the results of WES and panel NGS (38% vs 36%, respectively).

For 43 genes, pathogenic and likely pathogenic variants were reported in both WES and panel NGS results. Pathogenic (*P*) and likely pathogenic (*LP*) variants were found in 108 and 186 genes in panel NGS or WES tests, respectively, with no intersections (**Figure 3B**). For variants of uncertain significance (*VUS*), 83 genes were common, and 161 and 251 were specific for the panel NGS and WES tests, respectively (**Figure 3C**). In total, 281 and 515 genes had at least one *P*, *LP*, or *VUS* reported variant for the panel NGS and WES tests, respectively, and 120 genes hosted reported genetic variants common in both tests (**Supplementary Figure S1**).

### 3.2 Analysis of groups of patients.

For statistical analyses, the patients were grouped according to their clinical diagnoses by ICD10 sections (240 groups, **Supplementary File 3**). The biggest groups, each containing more than 100 genetically profiled patients, are listed in **Table 1**.

**Table 1:** ICD10 diagnostic sections containing more than 100 patients.

ICD10_section	WHO description	Diagnoses of the patients tested	Panel	WES	Total
E23	Hypofunction and other disorders of the pituitary gland	E23.0; E23.2; E23.3; E23.6; E23.7	574	112	686
E10	Type 1 diabetes mellitus	E10; E10.0; E10.1; E10.2; E10.3; E10.4; E10.6; E10.7; E10.8; E10.9	371	165	536
R73	Elevated blood glucose level	R73; R73.0; R73.9	408	2	410
E14	Unspecified diabetes mellitus	E14; E14.0; E14.7; E14.8; E14.9	392	7	399
E03	Other hypothyroidism	E03; E03.0; E03.1; E03.2; E03.8; E03.9	291	103	394
E34	Other endocrine disorders	E34; E34.3; E34.4; E34.5; E34.8; E34.9	230	128	358
E16	Other disorders of pancreatic internal secretion	E16.0; E16.1; E16.2; E16.4; E16.8; E16.9	248	97	345
E13	Other specified diabetes mellitus	E13; E13.2; E13.4; E13.7; E13.8; E13.9	297	10	307
E21	Hyperparathyroidism and other disorders of parathyroid gland	E21.0; E21.1; E21.2; E21.3; E21.4; E21.5	292	15	307
E66	Obesity	E66.0; E66.1; E66.8; E66.9	143	141	284
E25	Adrenogenital disorders	E25.0; E25.8; E25.9	261	21	282
E22	Hyperfunction of the pituitary gland	E22.0; E22.1; E22.8; E22.9	155	100	255
E27	Other disorders of the adrenal gland	E27; E27.0; E27.1; E27.3; E27.4; E27.5; E27.8; E27.9	207	32	239
E11	Type 2 diabetes mellitus	E11.2; E11.3; E11.4; E11.5; E11.6; E11.7; E11.8; E11.9	204	18	222
E83	Disorders of mineral metabolism	E83.3; E83.4; E83.5; E83.8; E83.9	194	11	205
E04	Other non-toxic goiter	E04.0; E04.1; E04.2	161	5	166
E31	Polyglandular dysfunction	E31; E31.0; E31.1; E31.8; E31.9	115	24	139
E30	Disorders of puberty, not elsewhere classified	E30; E30.0; E30.1; E30.8; E30.9	91	37	128

In **Table 1**, some ICD10 diagnosis sections have broad definitions and include the following specific diagnoses for the clinical group under investigation:

- for E03 *Other hypothyroidism*—E03.0 Congenital hypothyroidism with diffuse goitre, E03.1 Congenital hypothyroidism without goitre, E03.2 Hypothyroidism due to medicaments, E03.8 Other specified hypothyroidism, E03.9: Hypothyroidism, unspecified;
- for E04 *Other nontoxic goiter*—E04.0 Non-toxic diffuse goiter, E04.1 Non-toxic single thyroid nodule, E04.2 Non-toxic multinodular goiter;
- for E13 *Other* sp*ecified diabetes mellitus*—E13.2 Other specified diabetes mellitus with renal complications, E13.4 Other specified diabetes mellitus with neurological complications, E13.7 Other specified diabetes mellitus with multiple complications, E13.8 Other specified diabetes mellitus with unspecified complications, E13.9 Other specified diabetes mellitus without complications;
- for E16 *Other disorders of pancreatic internal secretion*—E16.0 Drug-induced hypoglycemia without coma, E16.1 Other hypoglycemia, E16.2 Hypoglycemia, unspecified, E16.4 Abnormal secretion of gastrin, E16.8 Other specified disorders of pancreatic internal secretion, E16.9 Disorder of pancreatic internal secretion, unspecified;
- for E23 *Hypofunction and other disorders of pituitary gland* —E23.0 Hypopituitarism, E23.2: Diabetes insipidus, E23.3 Hypothalamic dysfunction, not elsewhere classified, E23.6 Other disorders of pituitary gland, E23.7 Disorder of pituitary gland, unspecified;
- for E27 *Other disorders of adrenal gland*—E27.0 Other adrenocortical overactivity, E27.1 Primary adrenocortical insufficiency, E27.3 Drug-induced adrenocortical insufficiency, E27.4 Other and unspecified adrenocortical insufficiency, E27.5 Adrenomedullary hyperfunction, E27.8 Other specified disorders of adrenal gland, E27.9 Disorder of adrenal gland, unspecified;
- for E30 *Disorders of puberty, not elsewhere classified*—E30.0 Delayed puberty, E30.1 Precocious puberty, E30.8 Other disorders of puberty, E30.9 Disorder of puberty, unspecified;
- for E34 *Other endocrine disorders*—E34.3 Short stature, not elsewhere classified, E34.4 Constitutional tall stature, E34.5 Androgen resistance syndrome, E34.8 Other specified endocrine disorders, E34.9 Endocrine disorder, unspecified.

**Figure 4:**
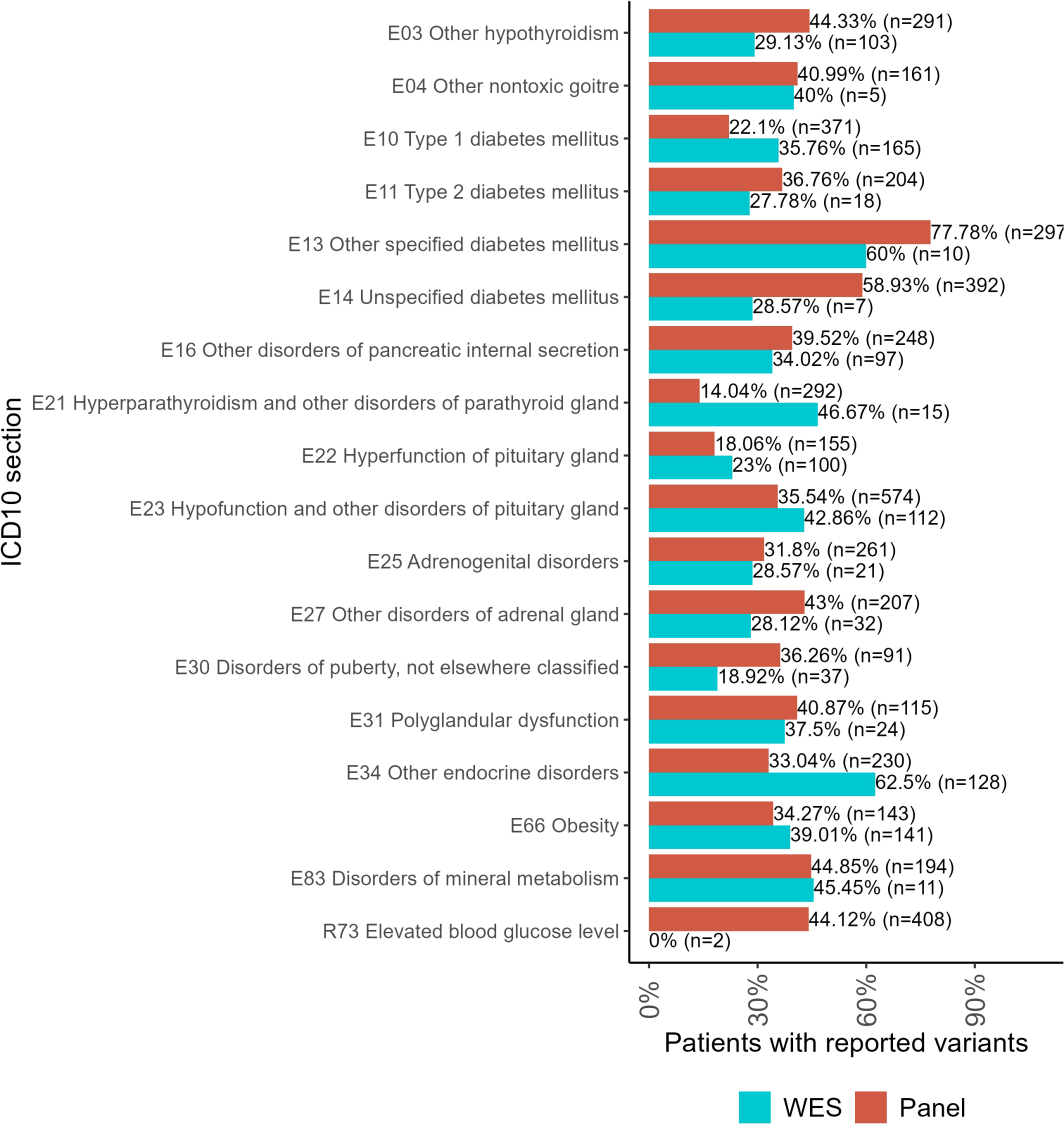
The proportion of patients with genetic variants classified as “pathogenic” (P), “likely pathogenic” (LP), or “uncertain significance” (VUS) in the results of panel NGS and WES tests for ICD10 diagnosis groups containing more than 100 genetically profiled patients with endocrine pathologies.

For the WES tests, the biggest proportion of reported variants was detected for the following patient groups (**Figure 4**): type 1 diabetes mellitus (E10), hyperparathyroidism and other disorders of parathyroid gland (E21), hyperfunction of pituitary gland (E22), hypofunction and other disorders of pituitary gland (E23), other endocrine disorders (E34), obesity (E66), and disorders of mineral metabolism (E83).

For panel NGS, the biggest proportion of reported variants was reported for the following groups: other hypothyroidism (E03); other non-toxic goiter (E04); type 2 diabetes mellitus (E11); other specified diabetes mellitus (E13); unspecified diabetes mellitus (E14); other disorders of pancreatic internal secretion (E16); adrenogenital disorders (E25); other disorders of the adrenal gland (E27); disorders of puberty, not elsewhere classified (E30); polyglandular dysfunction (E31); and elevated blood glucose level (R73).

**Figure 5:**
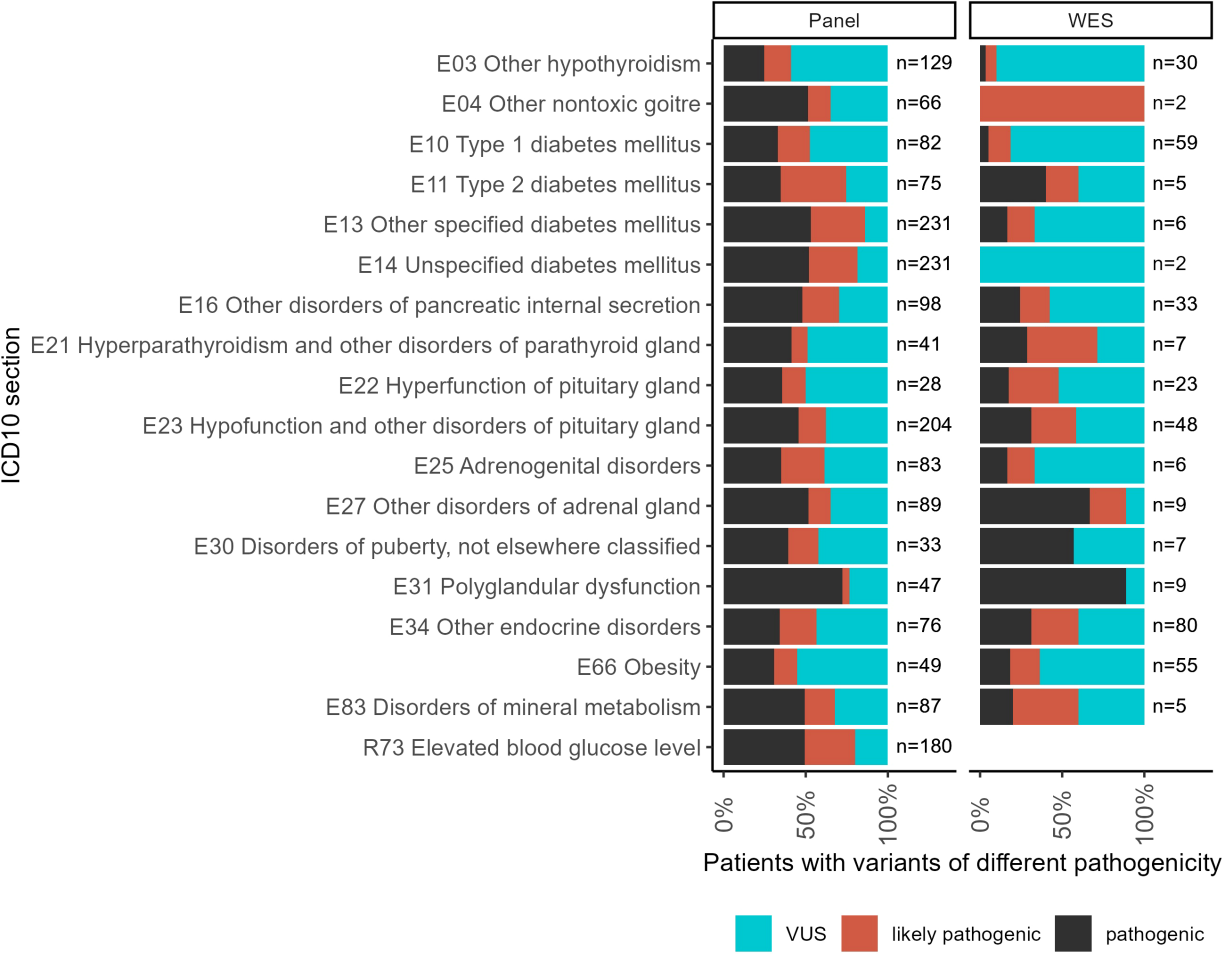
The proportion of patients with variants of different pathogenicity levels among all patients with reported variants for ICD10 diagnosis groups containing more than 100 genetically profiled patients with endocrine pathologies.

For each individual patient, the *pathogenicity level* was assessed by the highest pathogenicity score of their reported variants (**Figure 5**). Thus, the highest level (“pathogenic”) included patients with at least one pathogenic variant but who might have additional reported variants as well. Similarly, patients classified as having “likely pathogenic” variants could have other variants as well except for the “pathogenic” ones. The distribution of patients by pathogenicity level is shown in **Figure 5**.

**Figure 6:**
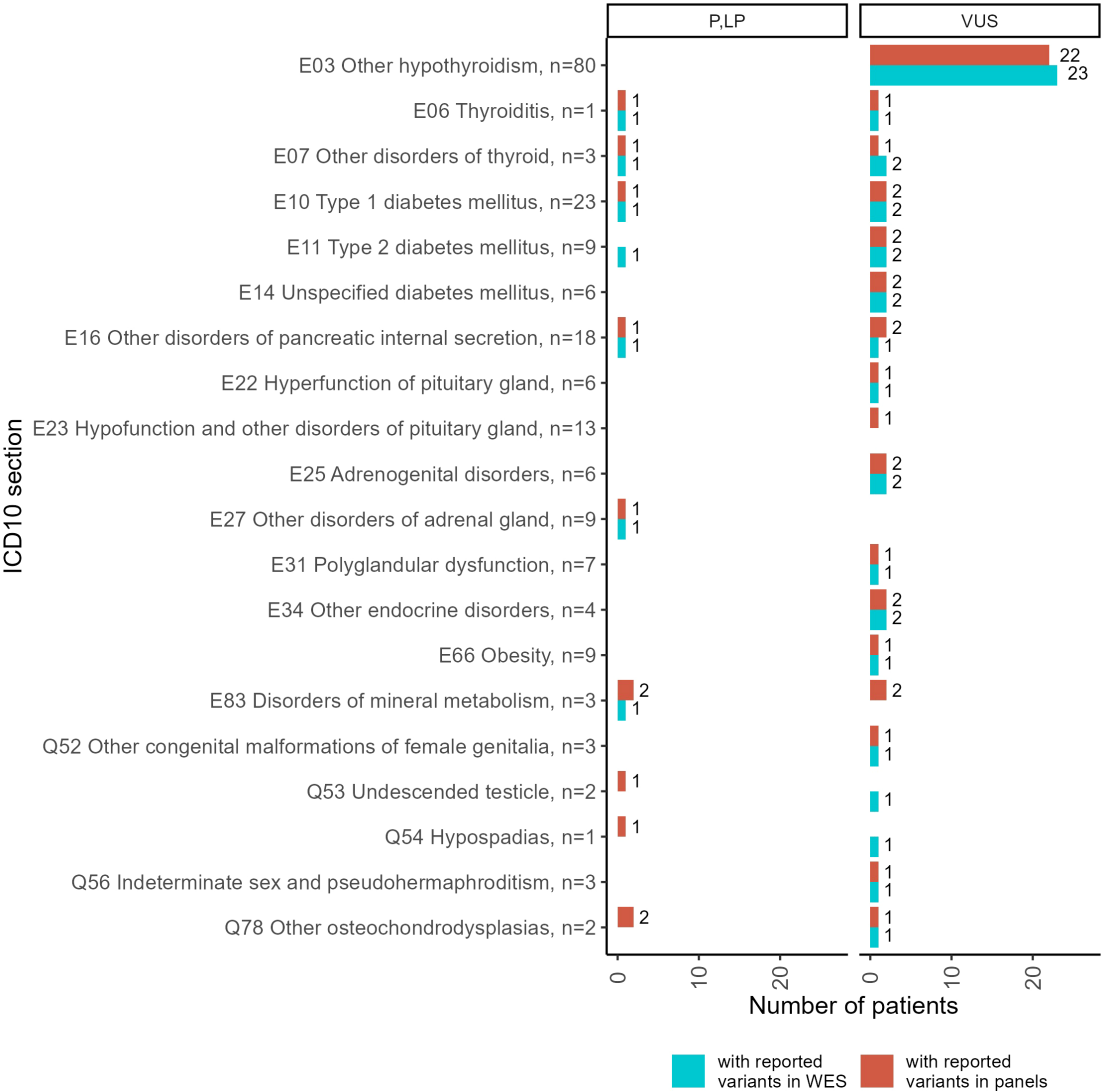
Statistics of patients with reported genetic variants classified as “pathogenic” (P), “likely pathogenic” (LP), or “uncertain significance” (VUS) in the results of double tests including panel NGS and WES, performed for 214 patients. Complete diagnoses of the patients tested are specified in **Supplementary File 3**.

Both panel NGS and WES profiles were available for 214 patients (**Figure 6**, **Supplementary File 3**). Thus, we compared the genetic variants reported in the same patients using alternative tests. In general, the WES results contained more reported variants than the panel NGS annotations. However, some variants were reported in the panel NGS results and then labeled as irrelevant to the patient’s condition in the WES tests. Because the geneticists subjected the patients to WES after panel NGS in cases of doubt when the first test could not adequately explain the patient’s phenotype, here we consider WES results as the gold standard for cases of such dual profiling.

A more detailed comparison of the molecular cases for the patients simultaneously profiled by panel NGS and WES including the distribution of gene mutation frequencies is given in **Supplementary File 4**.

We then compared the frequencies of *P* and *LP* variants in panel NGS and WES results. For this analysis, we excluded panel NGS results that were dismissed by WES tests for the same patients (eight patient cases).

**Figure 7:**
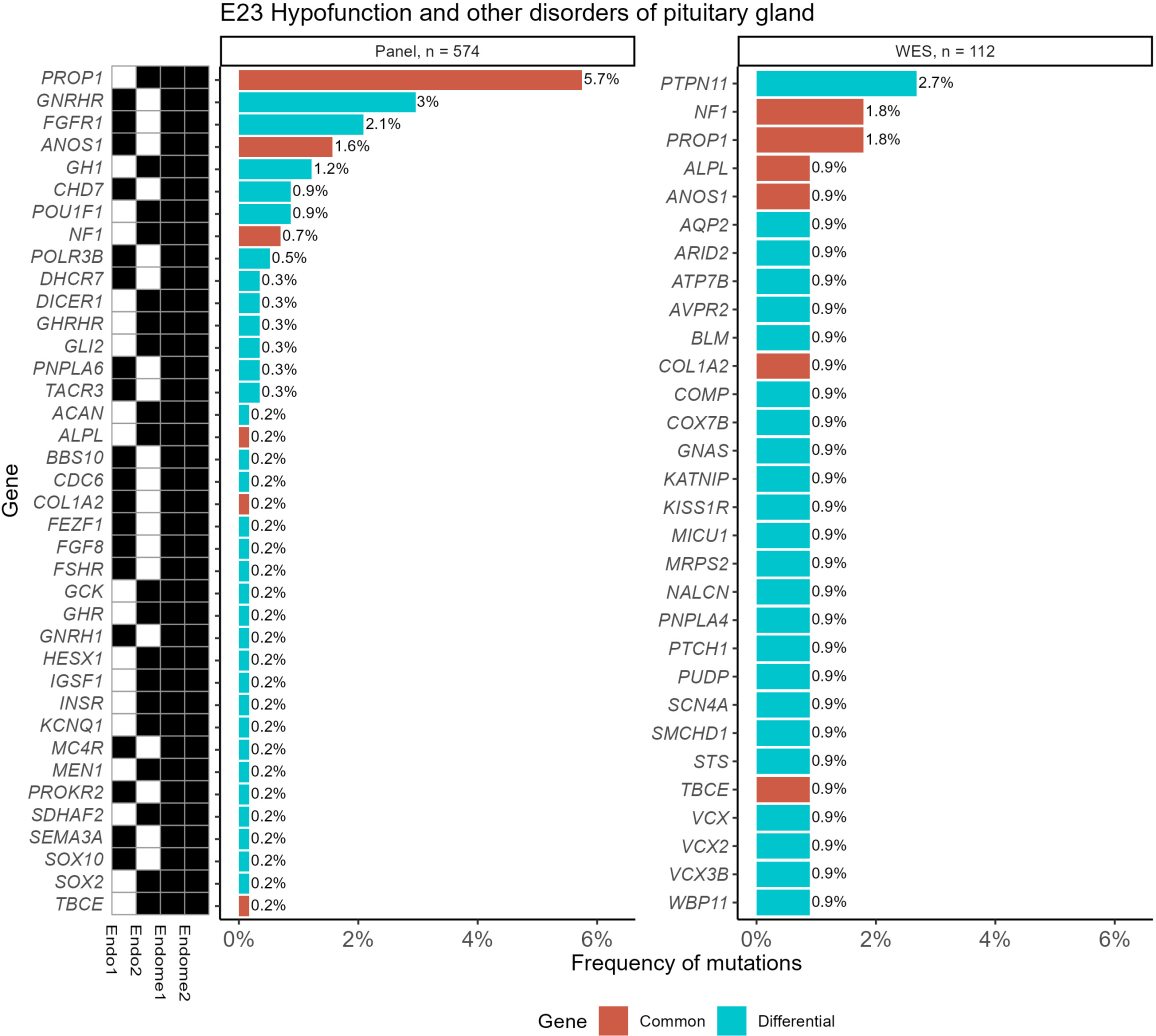
Frequencies of “pathogenic” (P) and “likely pathogenic” (LP) genetic variants for the ICD10 diagnosis group “E23 Hypofunction and other disorders of pituitary gland” identified using WES and panel NGS tests. Mutation frequency was calculated as the ratio of patients with gene mutations to the total number of patients in the group. Genes with pathogenic and likely pathogenic variants found in both panel NGS and WES tests are highlighted in orange (common items), otherwise shown in green (differential genes). The black marker shows whether the gene was included (black–yes, white–no) in the specific versions of the NGS panel used.

In **Figure 7**, such an analysis is exemplified for the ICD10 diagnosis group “E23 *Hypofunction and other disorders of pituitary gland*”. It can be seen that gene *PTPN11*, which was most frequently associated with the diagnosis “E34.3 *Short stature due to endocrine disorder*”, was also useful for the analysis of the E23 group.

For other ICD10 diagnosis groups containing more than 100 genetically profiled patients with endocrine pathologies, complete lists of genes hosting reported variants and mutation frequency statistics are given in **Supplementary File 5** for both WES and panel NGS tests.

**Figure 8:**
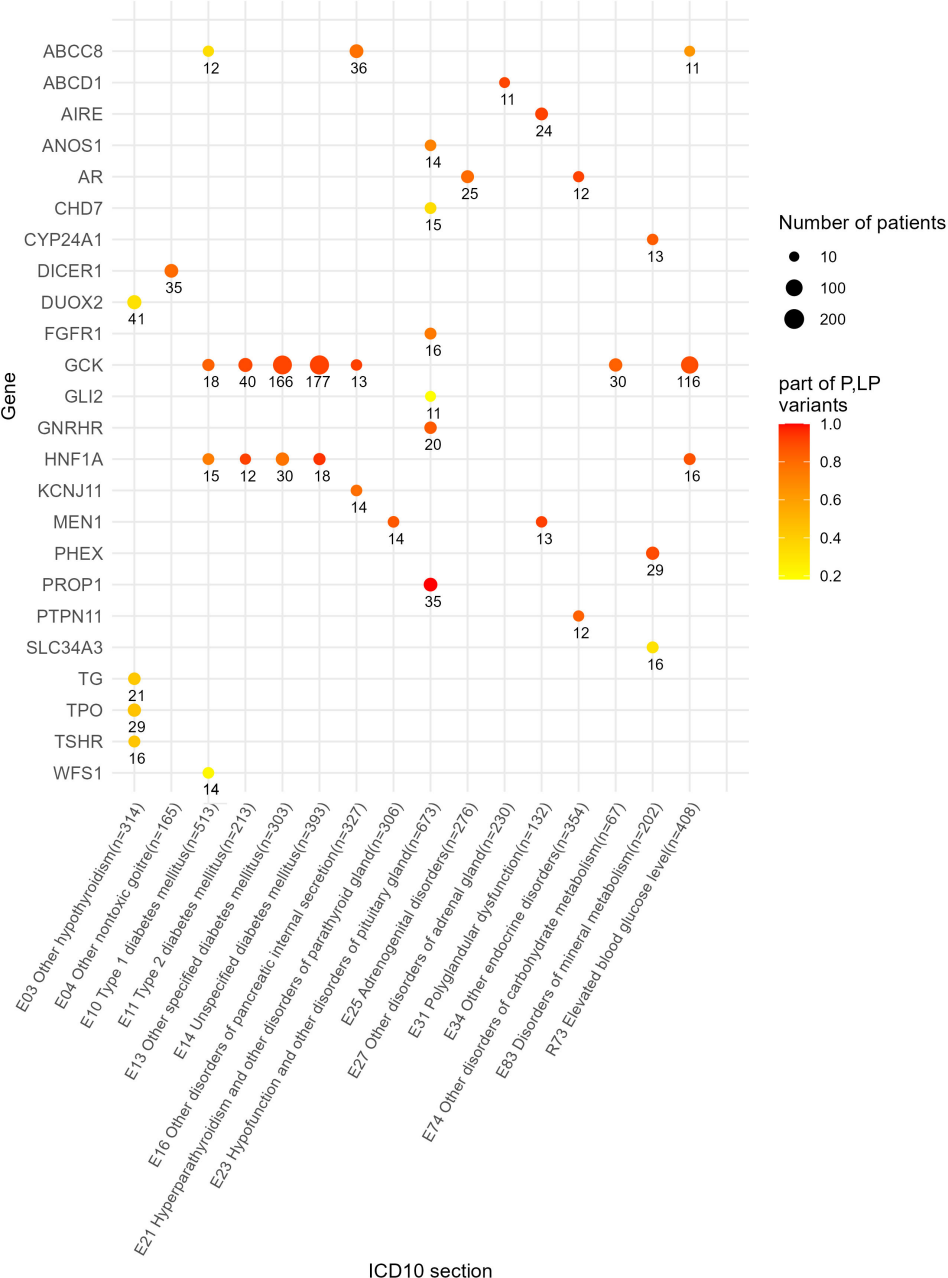
Genes with 10 times and greater occurrence in genetic reports in the whole patient cohort.

**Figure 9:**
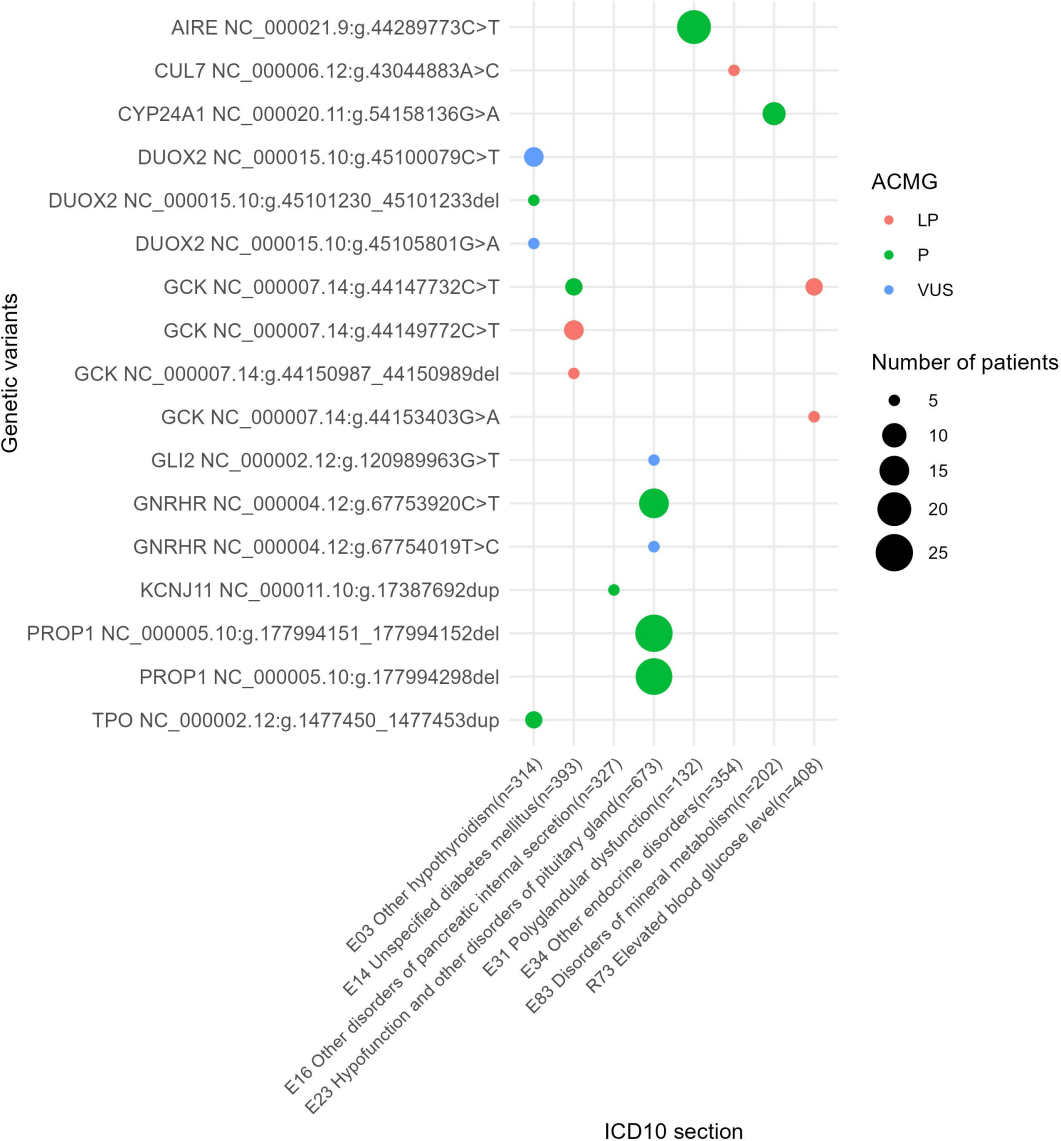
The most commonly reported genetic variants found in at least five patients under analysis.

We also identified a list of the most frequently mutated genes with a predominance of pathogenic and likely pathogenic reported variants that included genes *GCK* and *HNF1A* for diabetes mellitus phenotype or disorders of glucose metabolism (E10, E11, E13, E14, E74, an dR73); *KCNJ11*, *ABCC8* and *GCK* for other disorders of pancreatic internal secretion (E16); *AIRE* and *MEN1* for polyglandular dysfunction (E31); *AR* and *PTPN11* for the other endocrine disorders section including constitutional short stature; *GNRHR* and *PROP1* for hypofunction and other disorders of pituitary gland (E23); *DICER1* for other non-toxic goiter (E04); and *CYP24A1* and *PHEX* for disorders of mineral metabolism (**Figure 8**). In addition, 10 genes harbored relatively frequently reported variants that occurred in at least five patients under analysis (**Figure 9**).

**Figure 10:**
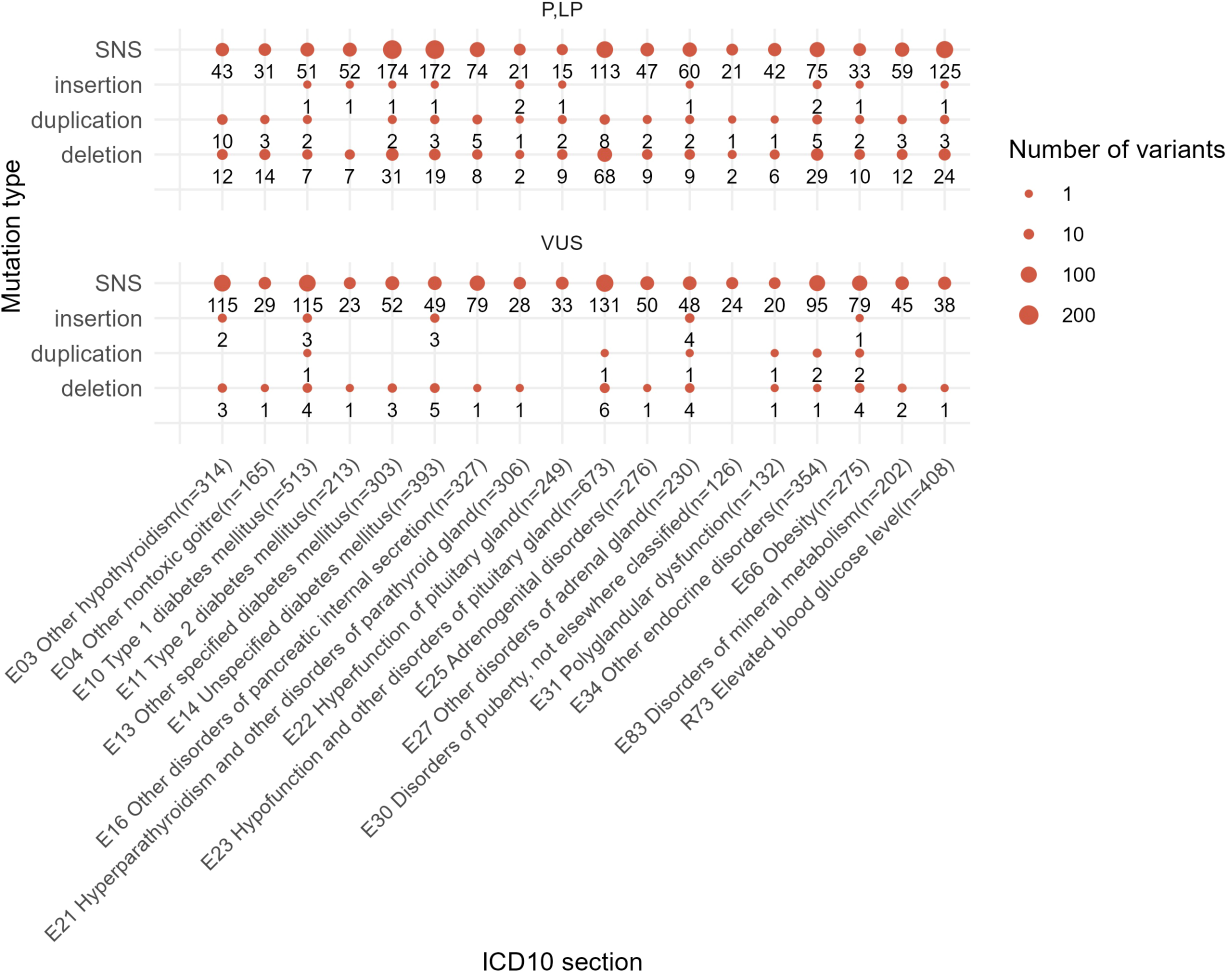
Statistics of different mutation types identified among the reported genetic variants in this study. One patient case may be included in several groups depending on the presence of mutations of a specific class.

In total, 1,184 out of 2,073 (57%) reported unique genetic variants were not described at the moment of NGS data interpretation by the geneticists. In the EndoGene database published here (https://doi.org/10.5281/zenodo.10894526), this is shown by the “*yes*” or “*no*” flags in the “Described in literature” column. The reported variants included 2,412 single nucleotide substitutions (SNS), 301 deletions, six insertions, 19 complex insertions and deletions, and 73 duplications. Out of them, four deletions and four duplications were long rearrangements involving at least several genes, as could be judged from the results of the WES analysis (**Figure 10**). In total, 2,811 variants (2,073 unique) were reported that could be classified as pathogenic, likely pathogenic, or VUS.

### 3.3 Next steps and limitations

Here, we present a database of genetic variants reported in patients with endocrine diseases and endocrine-related pathologies and in individuals at risk. We provided the ICD10 diagnosis codes for each patient and calculated the frequencies of genetic variants for the patients with diagnoses from the same ICD10 section. However, this article describes the raw data collection and does not intend to comprehensively interpret the data obtained. Thus, further statistical analysis will be needed to identify any associations of genetic variants with specific diagnoses.

Here, we report clinically relevant genetic variants in the standard HGVS format and classify associated diagnoses according to the ICD10 system, thus allowing this information to be converted and merged with other relevant knowledge bases.

## Data Availability

The datasets presented in this study can be found in online repositories. The names of the repository/repositories and accession number(s) can be found in the article/**Supplementary Material**.

## Supplementary material

Supplementary File 1 List of VEP - annotation fields for vcf files.

Supplementary File 2 Gene compositions of the NGS panels: Endo1, Endo2, Endome1, Endome2.

Supplementary File 3 Groups of patients by ICD10 diagnosis sections.

Supplementary File 4 Gene mutation frequencies for the patients who were simultaneously profiled by panel NGS and WES.

Supplementary File 5 Genes with frequencies of pathogenic and likely pathogenic variants for all groups of patients.

Supplementary Figure 1 The number of genes hosting genetic variants classified as being of pathogenic, likely pathogenic, and uncertain significance in the results of WES and panel NGS.
