# Supplementary material for: EndoGene database: reported genetic variants for 5,926 Russian patients diagnosed with endocrine disorders
Source: Front Endocrinol (Lausanne). 2025 Feb 18;16:1472754. doi: 10.3389/fendo.2025.1472754 (PMC11876052; doi:10.3389/fendo.2025.1472754)
Supplement: Supplementary File 1 — List of VEP - annotation fields for vcf files. [file DataSheet1.pdf]

VCF annotation fields:

##INFO=<ID=CSQ,Number=.,Type=String,Description="Consequence annotations from Ensembl VEP. Format: Allele|Consequence|IMPACT|SYMBOL|Gene|Feature_type|Feature|BIOTYPE|EXON|INTRON|HGVSc|HGVSp|cDNA_position|CDS_position|Protein_position|Amino_acids|Codons|Existing_variation|DISTANCE|STRAND|FLAGS|SYMBOL_SOURCE|HGNC_ID|ENSP|REFSEQ_MATCH|SOURCE|REFSEQ_OFFSET|NEAREST|SIFT|PolyPhen|HGVS_OFFSET|HGVSg|gnomADg_AF|gnomADg_AFR_AF|gnomADg_AMI_AF|gnomADg_AMR_AF|gnomADg_ASJ_AF|gnomADg_EAS_AF|gnomADg_FIN_AF|gnomADg_MID_AF|gnomADg_NFE_AF|gnomADg_OTH_AF|gnomADg_SAS_AF|CLIN_SIG|SOMATIC|PHENO|PUBMED|CADD_PHRED|CADD_RAW|BayesDel_addAF_pred|BayesDel_addAF_rankscore|BayesDel_addAF_score|BayesDel_noAF_pred|BayesDel_noAF_rankscore|BayesDel_noAF_score|MetaRNN_pred|MetaRNN_rankscore|MetaRNN_score|MutPred_AAchange|MutPred_Top5features|MutPred_protID|MutPred_rankscore|MutPred_score|SpliceAI_cutoff|SpliceAI_pred_DP_AG|SpliceAI_pred_DP_AL|SpliceAI_pred_DP_DG|SpliceAI_pred_DP_DL|SpliceAI_pred_DS_AG|SpliceAI_pred_DS_AL|SpliceAI_pred_DS_DG|SpliceAI_pred_DS_DL|SpliceAI_pred_SYMBOL|Mastermind_MMID3|Mastermind_counts|SpliceRegion|LoF|LoF_filter|LoF_flags|LoF_info">

##CADD_PHRED=PHRED-like scaled CADD score

##CADD_RAW=Raw CADD score

##BayesDel_addAF_pred=BayesDel_addAF_pred from dbNSFP file

##BayesDel_addAF_rankscore=BayesDel_addAF_rankscore from dbNSFP file

##BayesDel_addAF_score=BayesDel_addAF_score from dbNSFP file

##BayesDel_noAF_pred=BayesDel_noAF_pred from dbNSFP file

##BayesDel_noAF_rankscore=BayesDel_noAF_rankscore from dbNSFP file

##BayesDel_noAF_score=BayesDel_noAF_score from dbNSFP file

##MetaRNN_pred=MetaRNN_pred from dbNSFP file

##MetaRNN_rankscore=MetaRNN_rankscore from dbNSFP file

##MetaRNN_score=MetaRNN_score from dbNSFP file

##MutPred_AAchange=MutPred_AAchange from dbNSFP file

##MutPred_Top5features=MutPred_Top5features from dbNSFP file

##MutPred_protID=MutPred_protID from dbNSFP file

##MutPred_rankscore=MutPred_rankscore from dbNSFP file

##MutPred_score=MutPred_score from dbNSFP file

##SpliceAI_cutoff=Flag if delta score pass the cutoff (PASS) or if it does not (FAIL)

##SpliceAI_pred_DP_AG=SpliceAI predicted effect on splicing. Delta position for acceptor gain

##SpliceAI_pred_DP_AL=SpliceAI predicted effect on splicing. Delta position for acceptor loss

##SpliceAI_pred_DP_DG=SpliceAI predicted effect on splicing. Delta position for donor gain

##SpliceAI_pred_DP_DL=SpliceAI predicted effect on splicing. Delta position for donor loss

##SpliceAI_pred_DS_AG=SpliceAI predicted effect on splicing. Delta score for acceptor gain

##SpliceAI_pred_DS_AL=SpliceAI predicted effect on splicing. Delta score for acceptor loss

##SpliceAI_pred_DS_DG=SpliceAI predicted effect on splicing. Delta score for donor gain

##SpliceAI_pred_DS_DL=SpliceAI predicted effect on splicing. Delta score for donor loss

##SpliceAI_pred_SYMBOL=SpliceAI gene symbol

##Mastermind_MMID3=Mastermind MMID3 variant identifier(s), as gene:key. Link to the Genomenon Mastermind Genomic Search Engine.

##Mastermind_counts=Mastermind number of citations in the medical literature. Output includes three unique counts: MMCNT1|MMCNT2|MMCNT3. MMCNT1 - Count of Mastermind articles with cDNA matches for this specific variant; MMCNT2 - Count of Mastermind articles with variants either explicitly matching at the cDNA level or given only at protein level; MMCNT3 - Count of Mastermind articles including other DNA-level variants resulting in the same amino acid change.

##SpliceRegion=SpliceRegion predictions

##LoF=Loss-of-function annotation (HC = High Confidence; LC = Low Confidence)

##LoF_filter=Reason for LoF not being HC

##LoF_flags=Possible warning flags for LoF

##LoF_info=Info used for LoF annotation
