# Supplementary material for: EndoGene database: reported genetic variants for 5,926 Russian patients diagnosed with endocrine disorders
Source: Front Endocrinol (Lausanne). 2025 Feb 18;16:1472754. doi: 10.3389/fendo.2025.1472754 (PMC11876052; doi:10.3389/fendo.2025.1472754)
Supplement: Supplementary File 4 — Gene mutation frequencies for the patients who were simultaneously profiled by panel NGS and WES. [file DataSheet4.pdf]

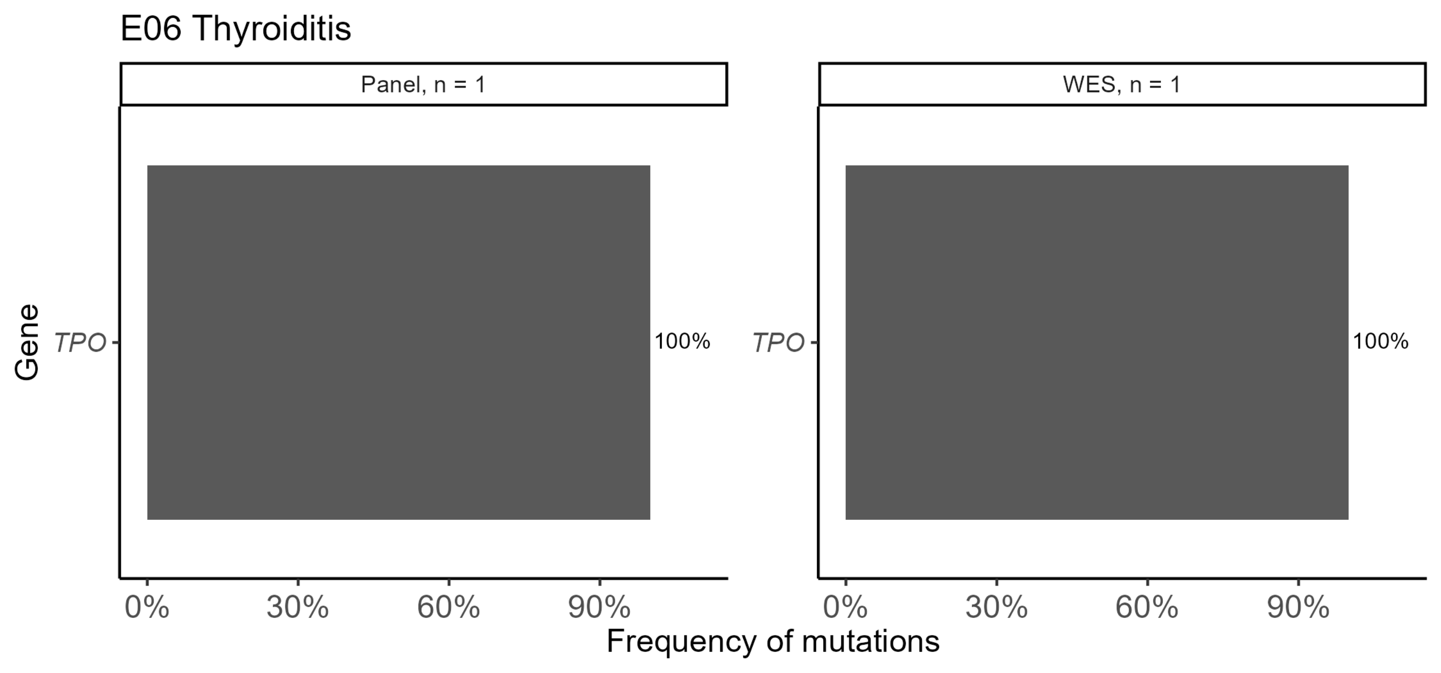
E06 Thyroiditis
Panel, n = 1
WES, n = 1
Gene
TPO
TPO
100%
100%
0%
30%
60%
90%
0%
30%
60%
90%
Frequency of mutations

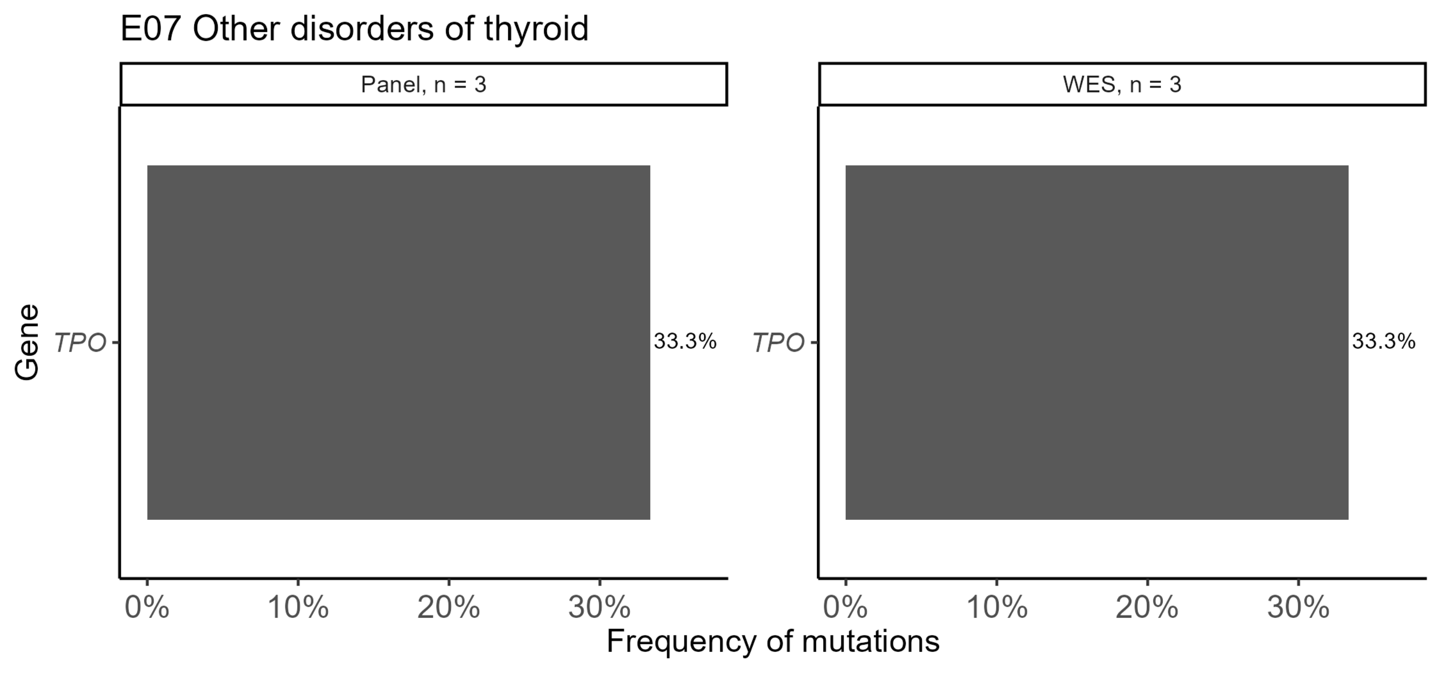
E07 Other disorders of thyroid
Panel, n = 3
WES, n = 3
Gene
TPO
33.3%
TPO
33.3%
0%
10%
20%
30%
0%
10%
20%
30%
Frequency of mutations

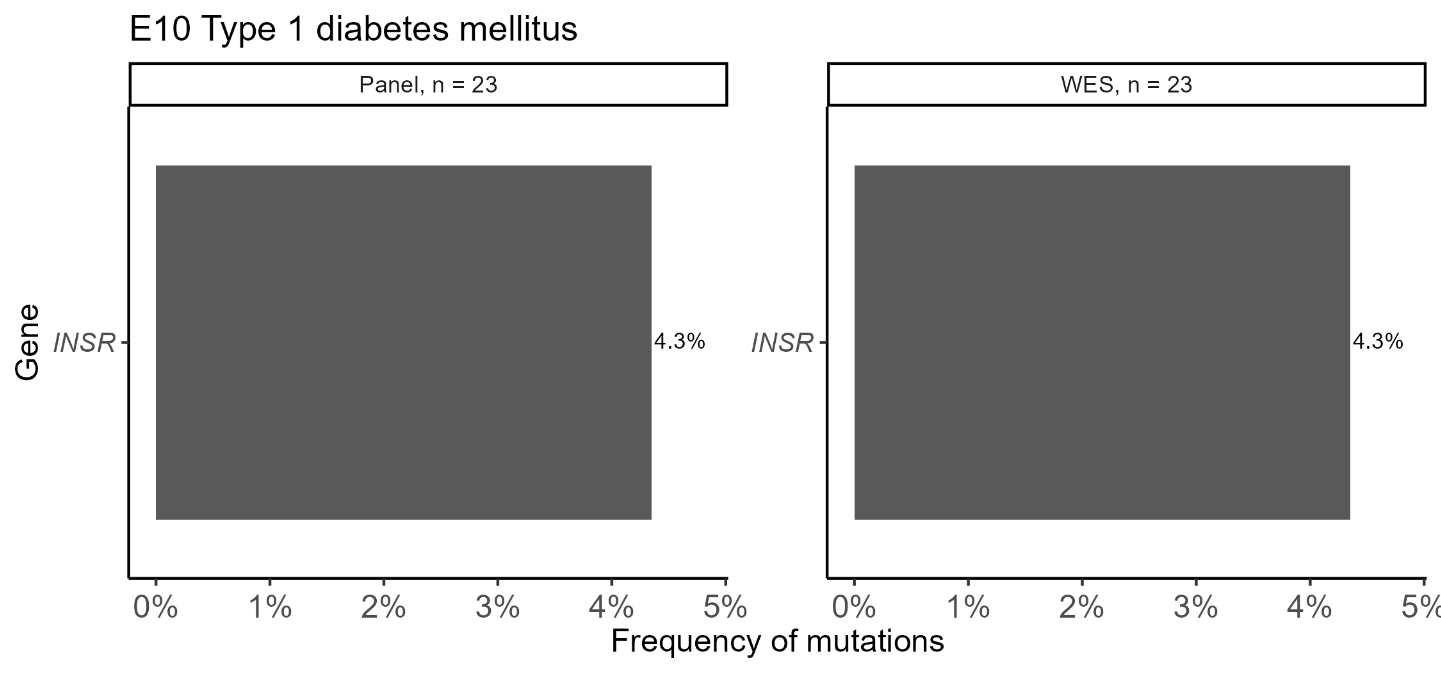

E10 Type 1 diabetes mellitus
Panel, n = 23
WES, n = 23
Gene
INSR
4.3%
INSR
4.3%
0%
1%
2%
3%
4%
5%
0%
1%
2%
3%
4%
5%
Frequency of mutations

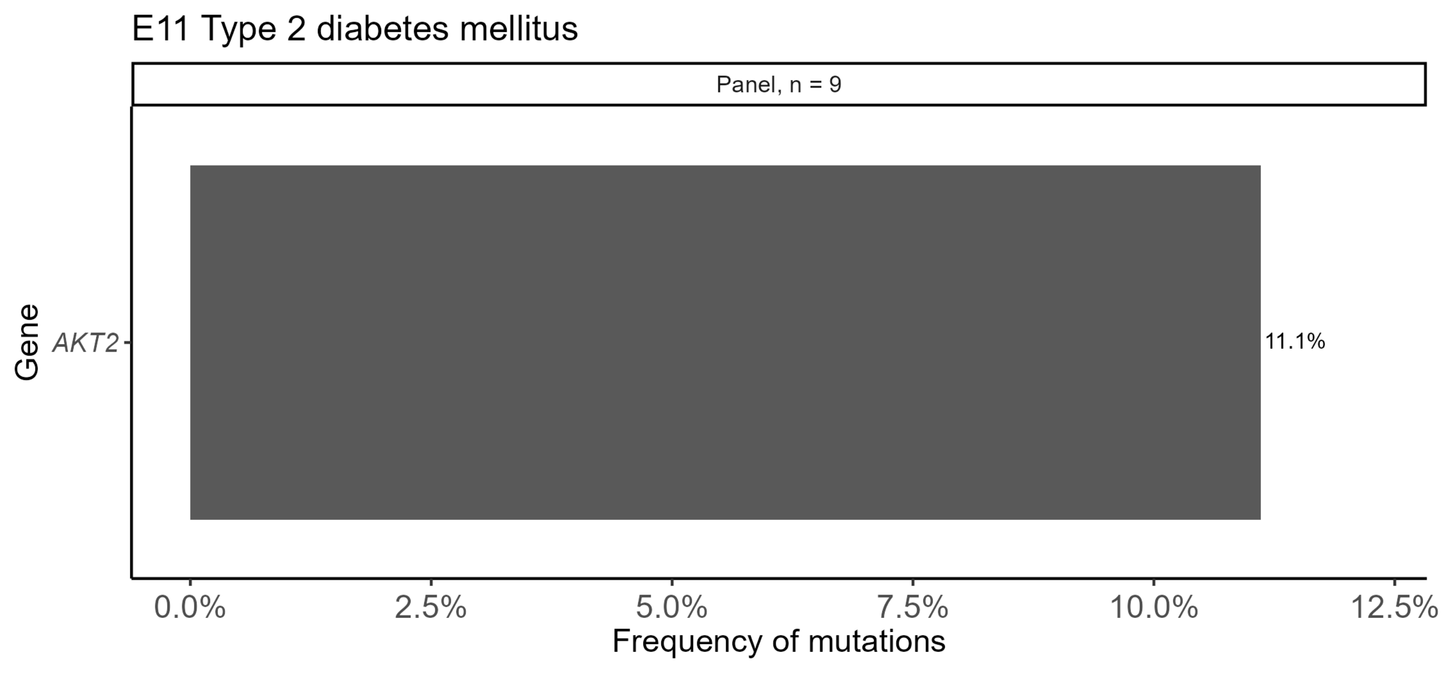
E11 Type 2 diabetes mellitus
Panel, n = 9
Gene
AKT2
11.1%
0.0%
2.5%
5.0%
7.5%
10.0%
12.5%
Frequency of mutations

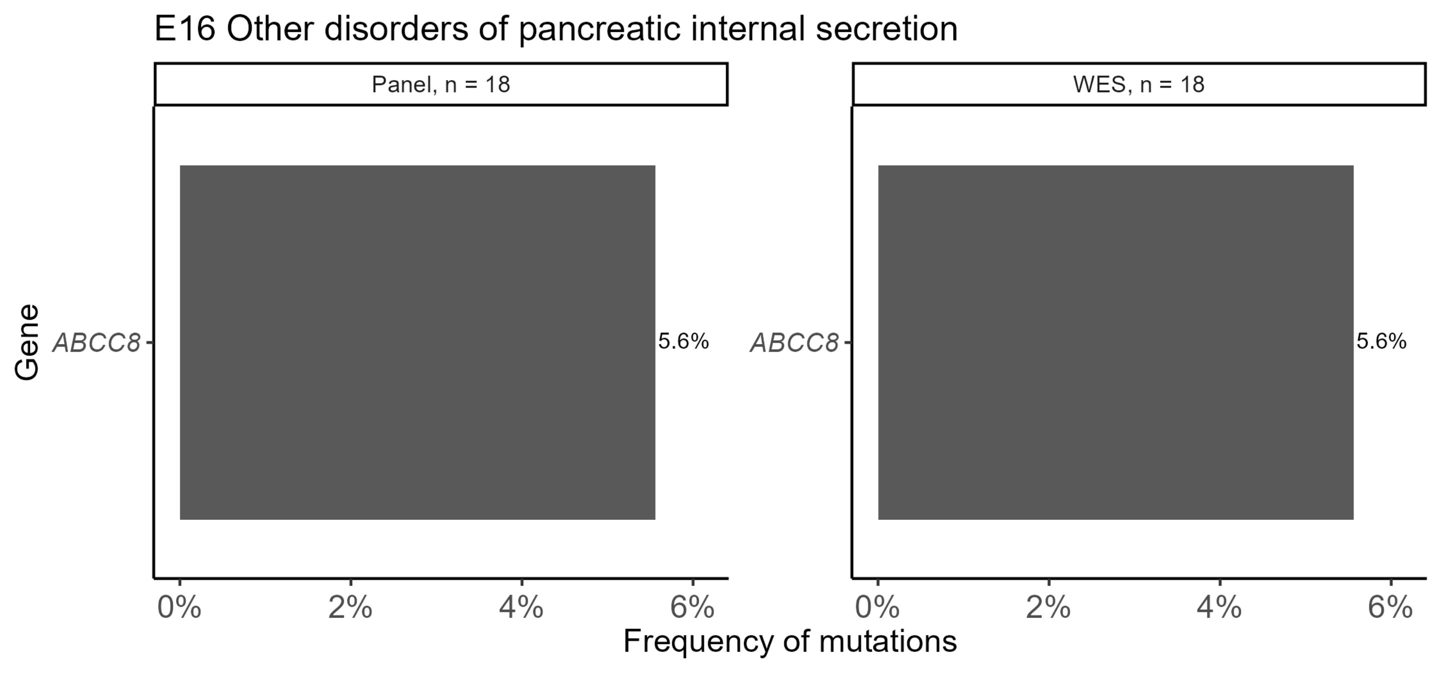
E16 Other disorders of pancreatic internal secretion
Panel, n = 18
WES, n = 18
Gene
ABCC8
5.6%
ABCC8
5.6%
0%
2%
4%
6%
0%
2%
4%
6%
Frequency of mutations

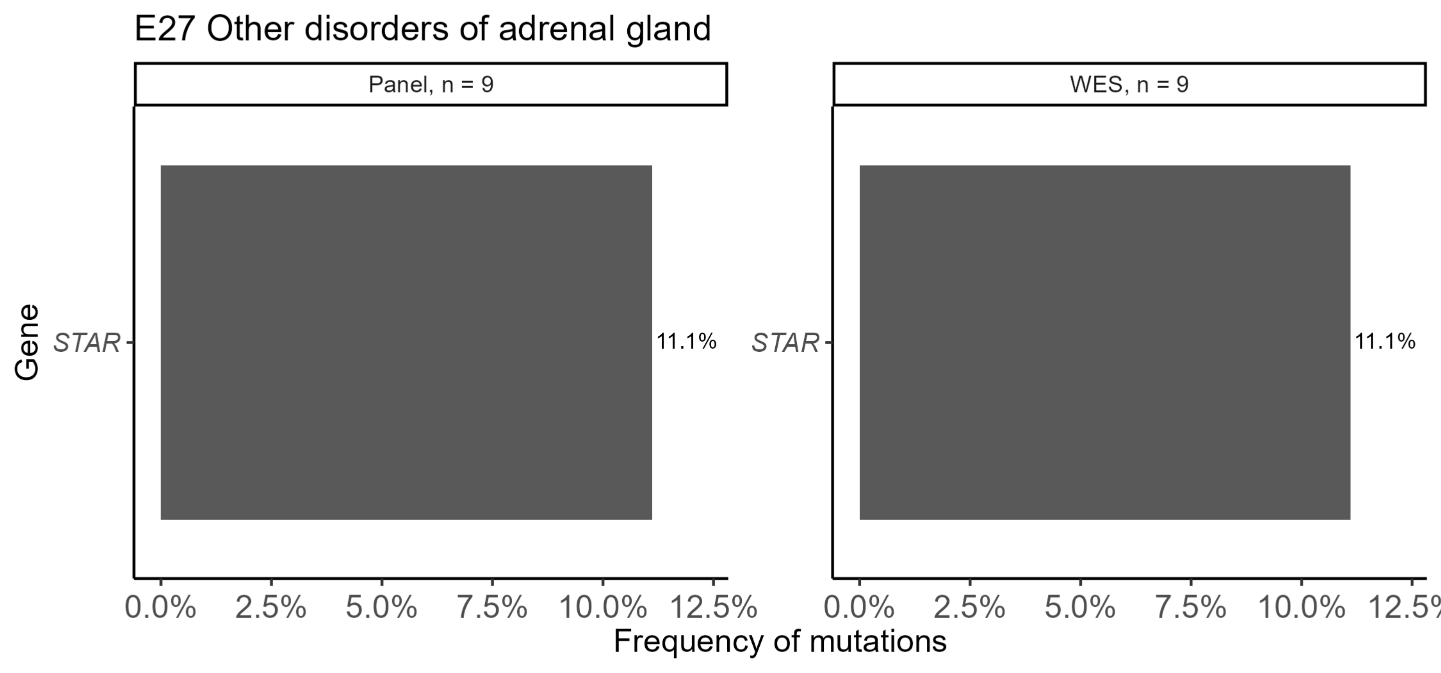
E27 Other disorders of adrenal gland
Panel, n = 9
WES, n = 9
Gene
STAR
11.1%
STAR
11.1%
0.0%
2.5%
5.0%
7.5%
10.0%
12.5%
Frequency of mutations

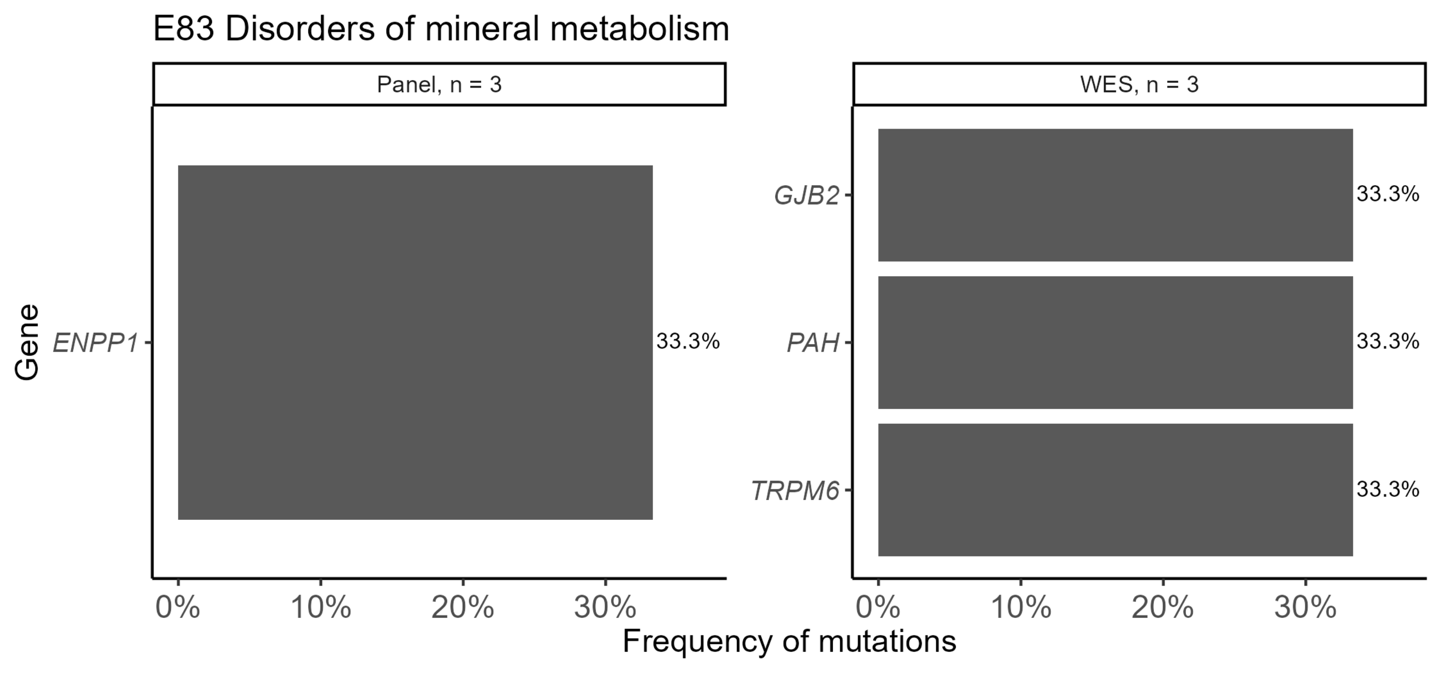

E83 Disorders of mineral metabolism
Panel, n = 3
WES, n = 3
Gene
ENPP1
33.3%
GJB2
PAH
TRPM6
33.3%
33.3%
33.3%
0%
10%
20%
30%
0%
10%
20%
30%
Frequency of mutations

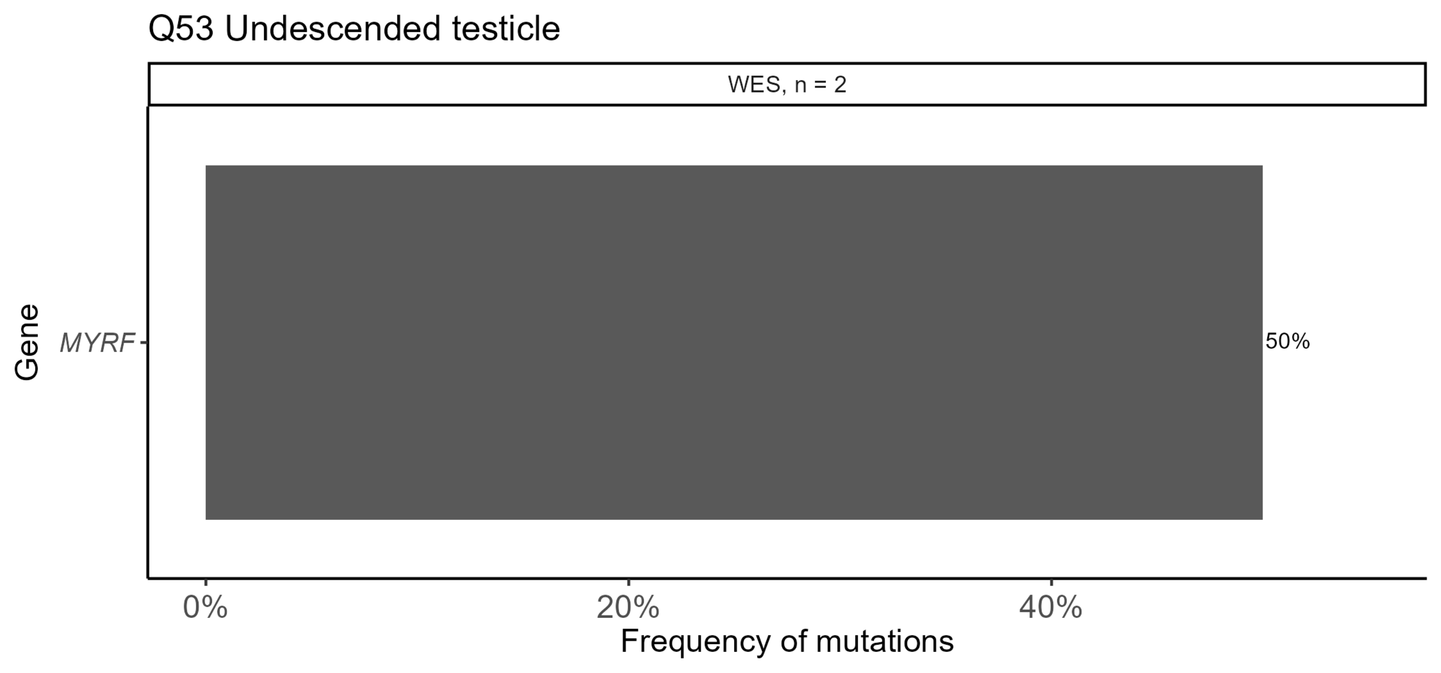
Q53 Undescended testicle
WES, n = 2
Gene
MYRF
50%
0%
20%
40%
Frequency of mutations

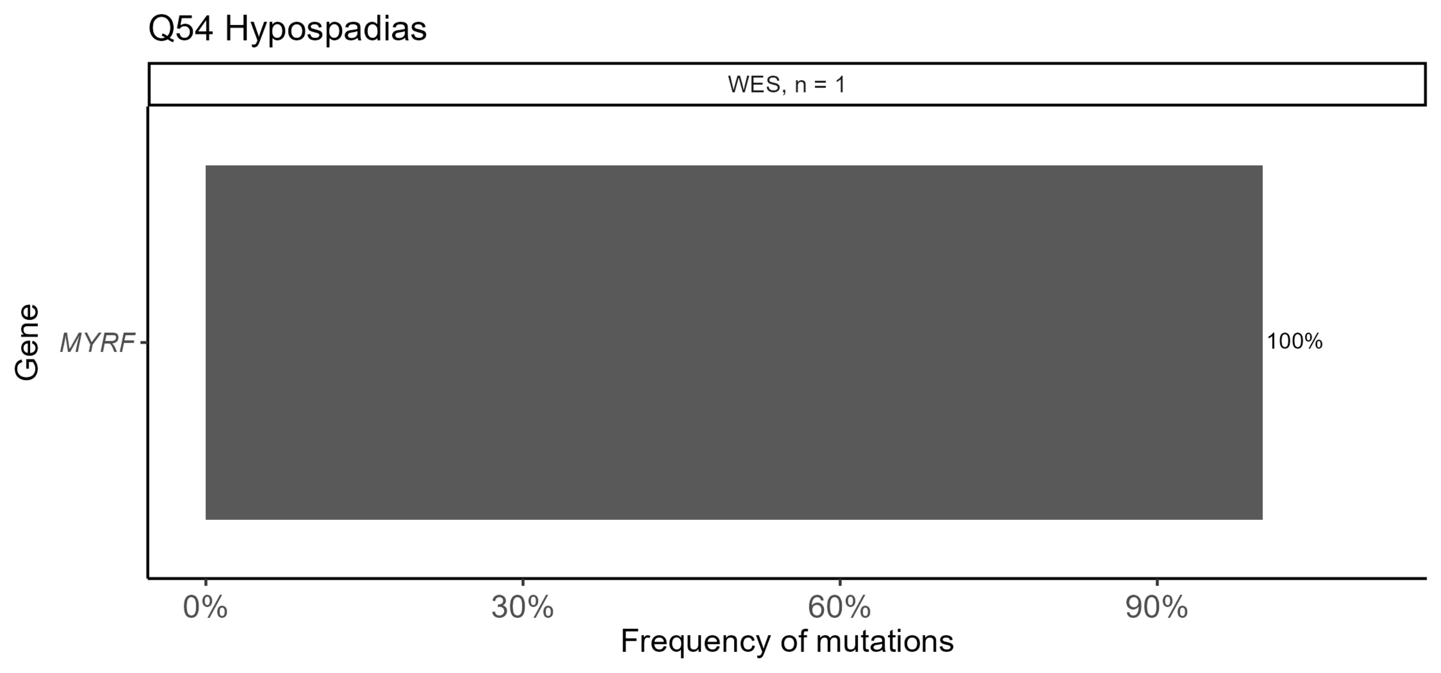
Q54 Hypospadias
WES, n = 1
Gene
MYRF
100%
0%
30%
60%
90%
Frequency of mutations

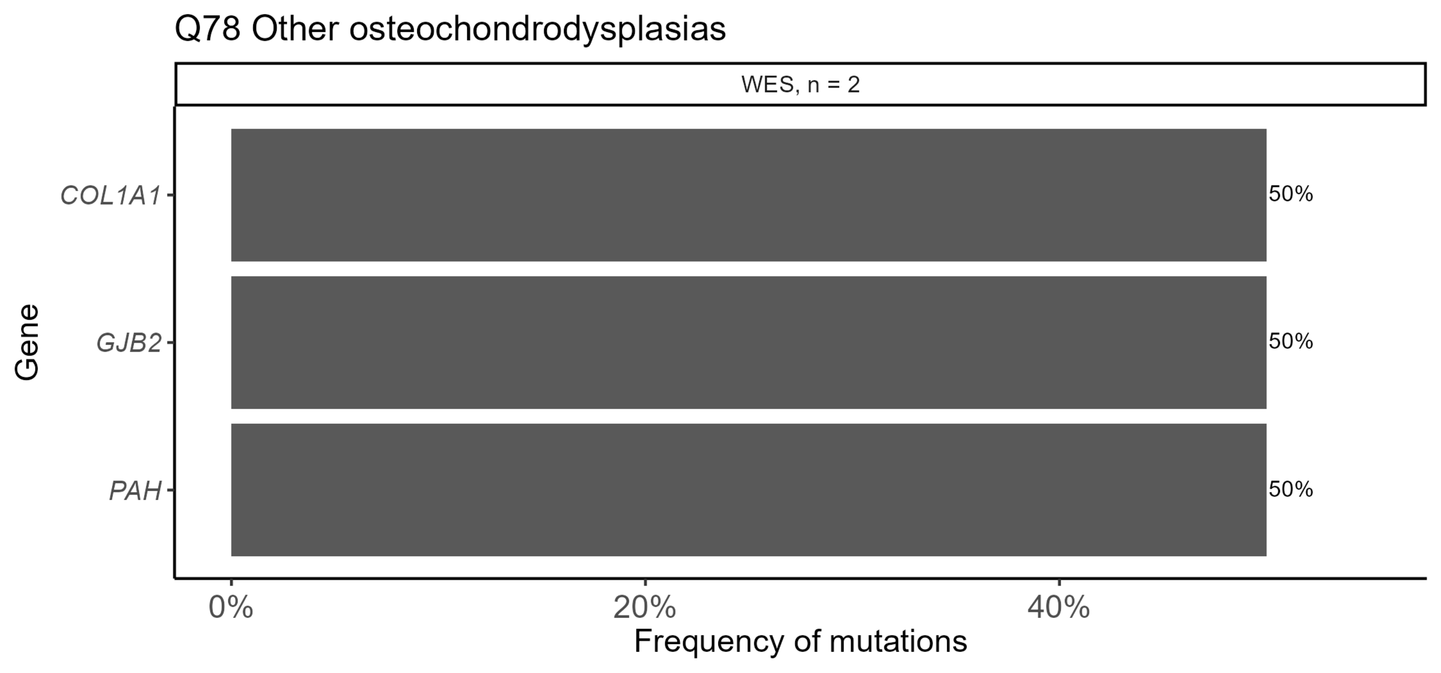
Q78 Other osteochondrodysplasias
WES, n = 2
COL1A1
GJB2
PAH
50%
50%
50%
Gene
0%
20%
40%
Frequency of mutations
