# Supplementary material for: EndoGene database: reported genetic variants for 5,926 Russian patients diagnosed with endocrine disorders
Source: Front Endocrinol (Lausanne). 2025 Feb 18;16:1472754. doi: 10.3389/fendo.2025.1472754 (PMC11876052; doi:10.3389/fendo.2025.1472754)
Supplement: Supplementary File 5 — Genes with frequencies of pathogenic and likely pathogenic variants for all groups of patients. [file DataSheet5.pdf]

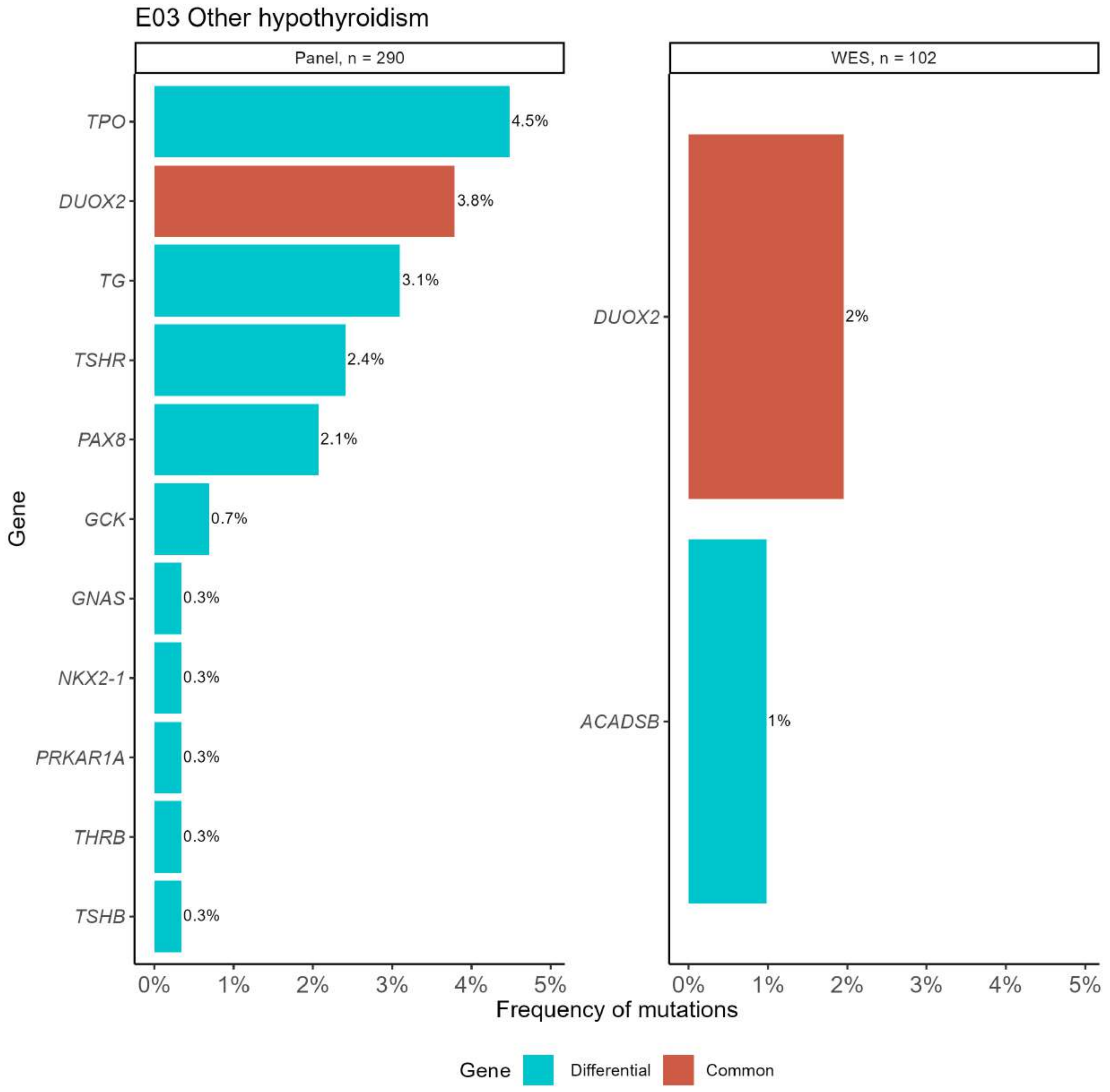

E03 Other hypothyroidism
Panel, n = 290
WES, n = 102
TPO
DUOX2
TG
TSHR
PAX8
GCK
GNAS
NKX2-1
PRKAR1A
THRB
TSHB
4.5%
3.8%
3.1%
2.4%
2.1%
0.7%
0.3%
0.3%
0.3%
0.3%
0.3%
DUOX2
ACADSB
2%
1%
Gene
0%
1%
2%
3%
4%
5%
Frequency of mutations
Gene
Differential
Common

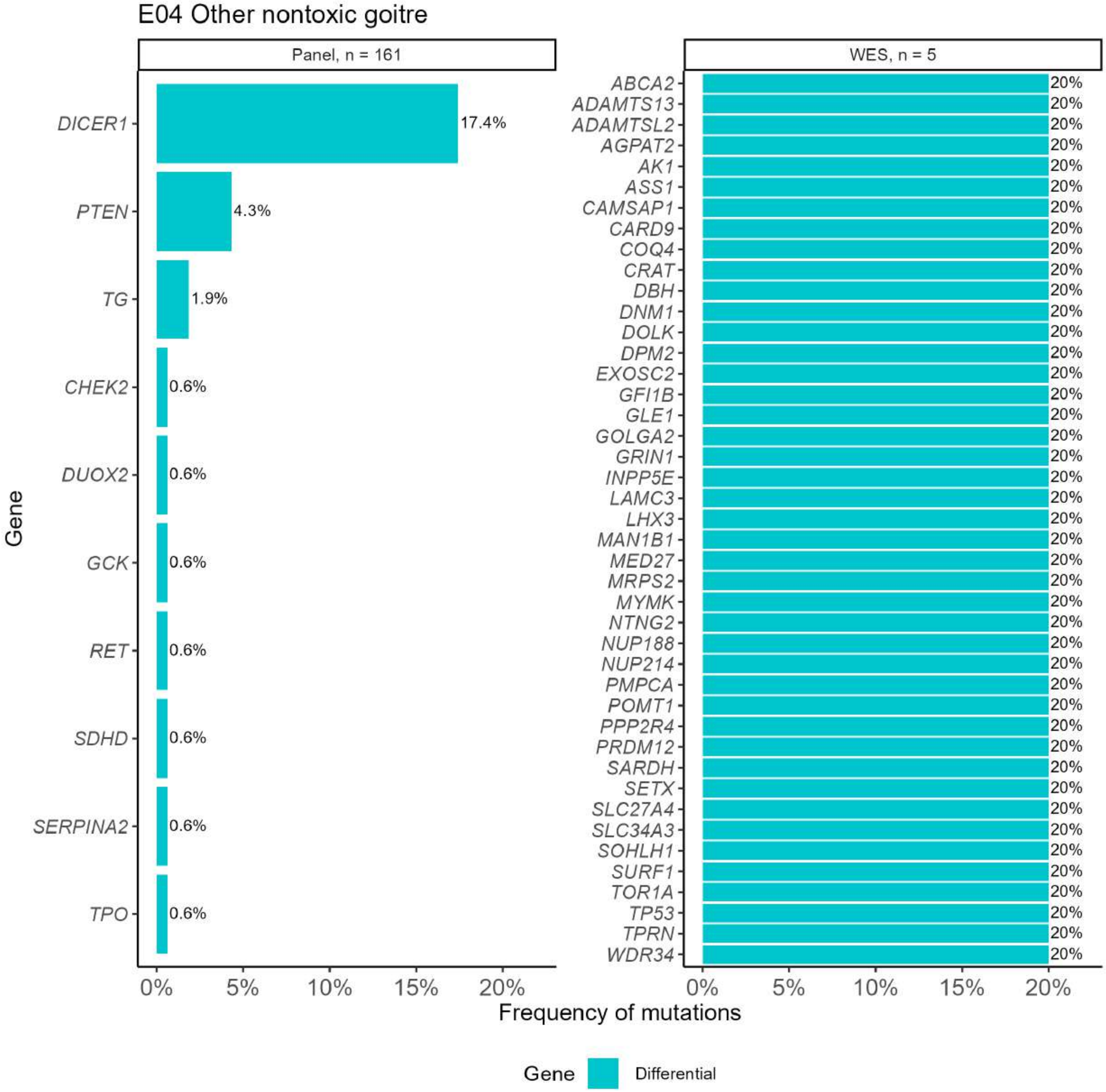
E04 Other nontoxic goitre
Panel, n = 161
WES, n = 5
Gene
DICER1 17.4%
PTEN 4.3%
TG 1.9%
CHEK2 0.6%
DUOX2 0.6%
GCK 0.6%
RET 0.6%
SDHD 0.6%
SERPINA2 0.6%
TPO 0.6%
ABCA2 20%
ADAMTS13 20%
ADAMTSL2 20%
AGPAT2 20%
AK1 20%
ASS1 20%
CAMSAP1 20%
CARD9 20%
COQ4 20%
CRAT 20%
DBH 20%
DNM1 20%
DOLK 20%
DPM2 20%
EXOSC2 20%
GFI1B 20%
GLE1 20%
GOLGA2 20%
GRIN1 20%
INPP5E 20%
LAMC3 20%
LHX3 20%
MAN1B1 20%
MED27 20%
MRPS2 20%
MYMK 20%
NTNG2 20%
NUP188 20%
NUP214 20%
PMPCA 20%
POMT1 20%
PPP2R4 20%
PRDM12 20%
SARDH 20%
SETX 20%
SLC27A4 20%
SLC34A3 20%
SOHLH1 20%
SURF1 20%
TOR1A 20%
TP53 20%
TPRN 20%
WDR34 20%
0% 5% 10% 15% 20%
Frequency of mutations
Gene Differential

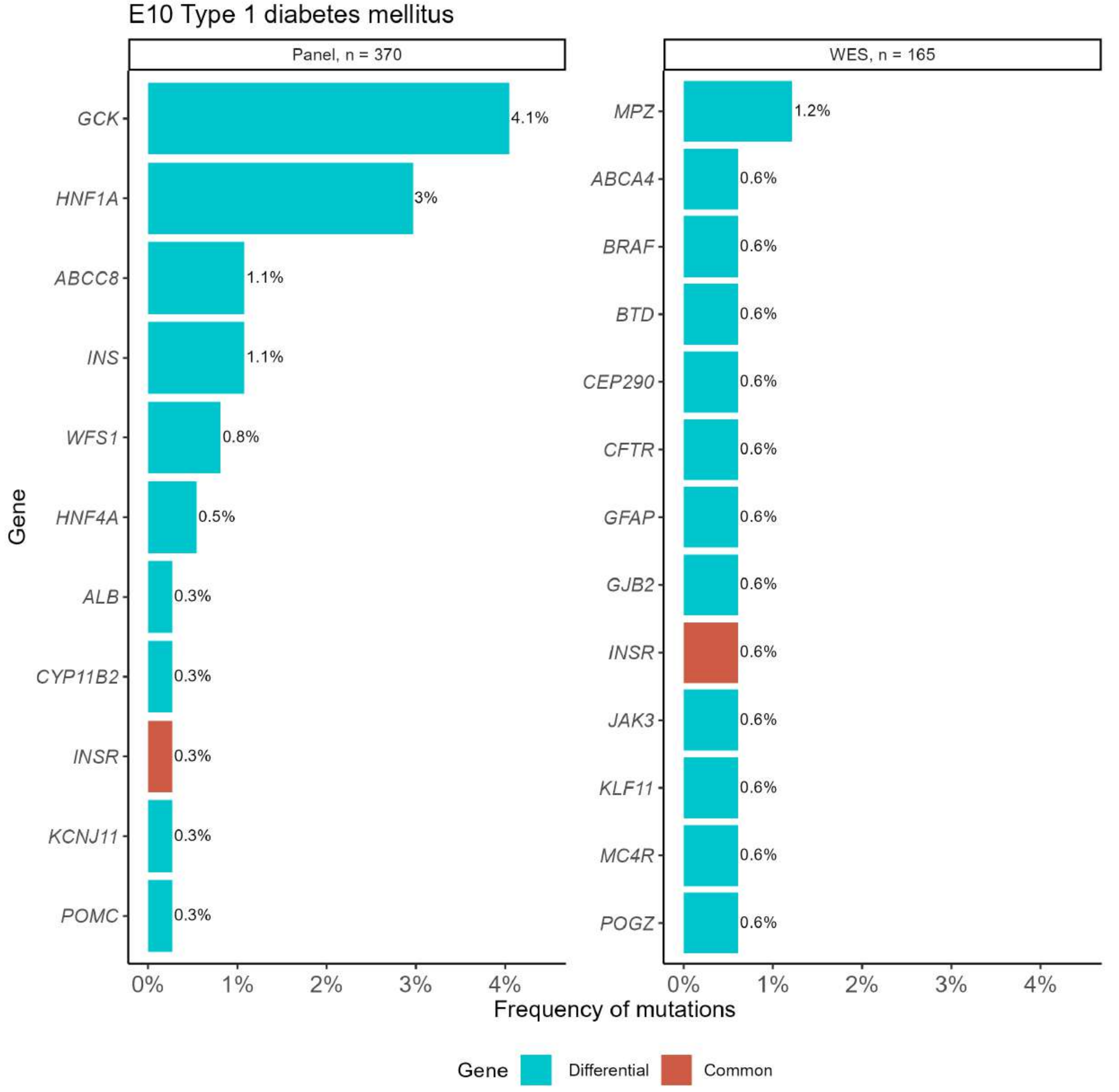

E10 Type 1 diabetes mellitus
Panel, n = 370
WES, n = 165
GCK
HNF1A
ABCC8
INS
WFS1
HNF4A
ALB
CYP11B2
INSR
KCNJ11
POMC
4.1%
3%
1.1%
1.1%
0.8%
0.5%
0.3%
0.3%
0.3%
0.3%
0.3%
MPZ
ABCA4
BRAF
BTD
CEP290
CFTR
GFAP
GJB2
INSR
JAK3
KLF11
MC4R
POGZ
1.2%
0.6%
0.6%
0.6%
0.6%
0.6%
0.6%
0.6%
0.6%
0.6%
0.6%
0.6%
0.6%
Gene
0%
1%
2%
3%
4%
Frequency of mutations
Gene
Differential
Common

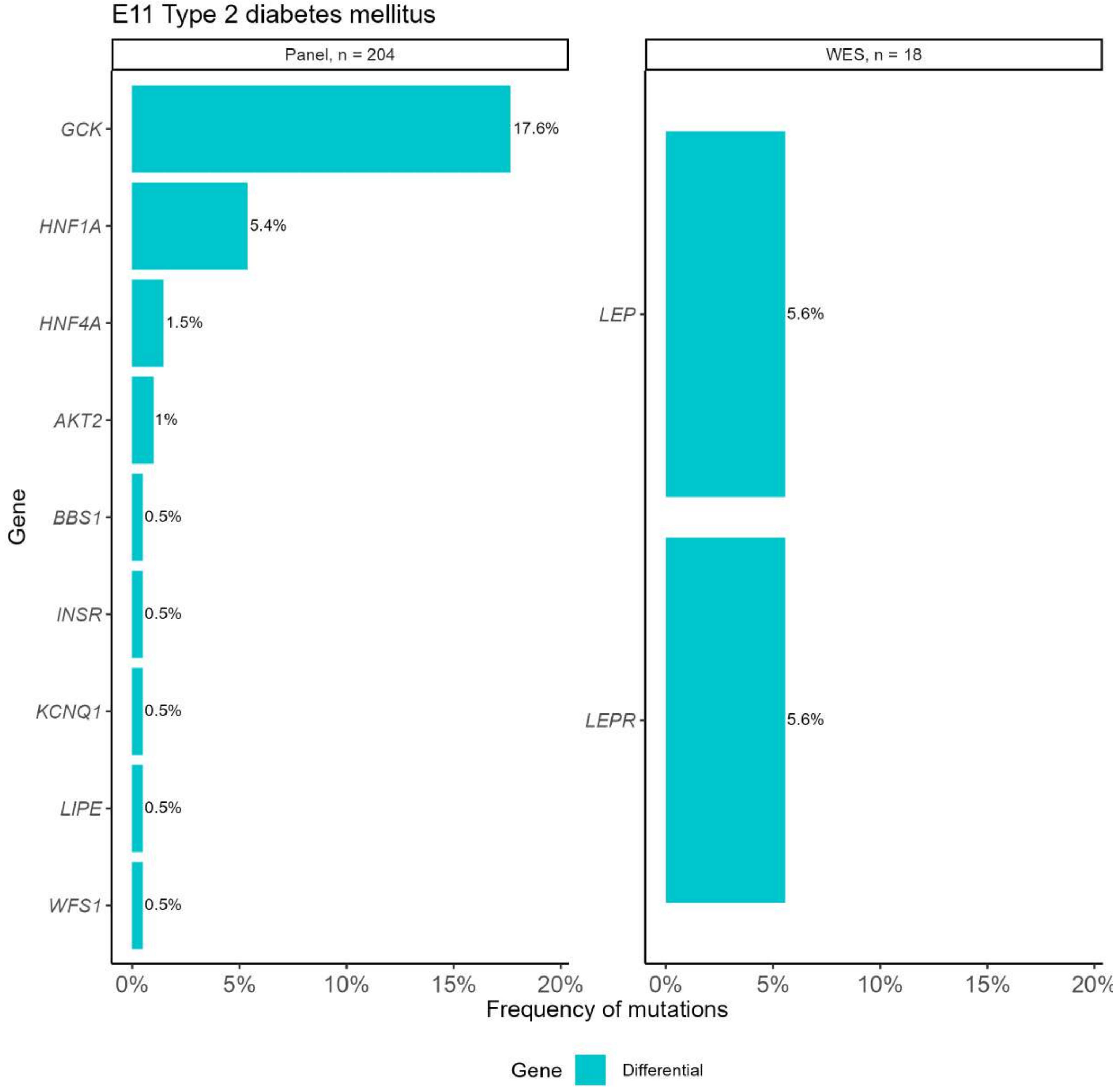
E11 Type 2 diabetes mellitus
Panel, n = 204
WES, n = 18
GCK
HNF1A
HNF4A
AKT2
BBS1
INSR
KCNQ1
LIPE
WFS1
17.6%
5.4%
1.5%
1%
0.5%
0.5%
0.5%
0.5%
0.5%
LEP
LEPR
5.6%
5.6%
Gene
0%
5%
10%
15%
20%
Frequency of mutations
Gene
Differential

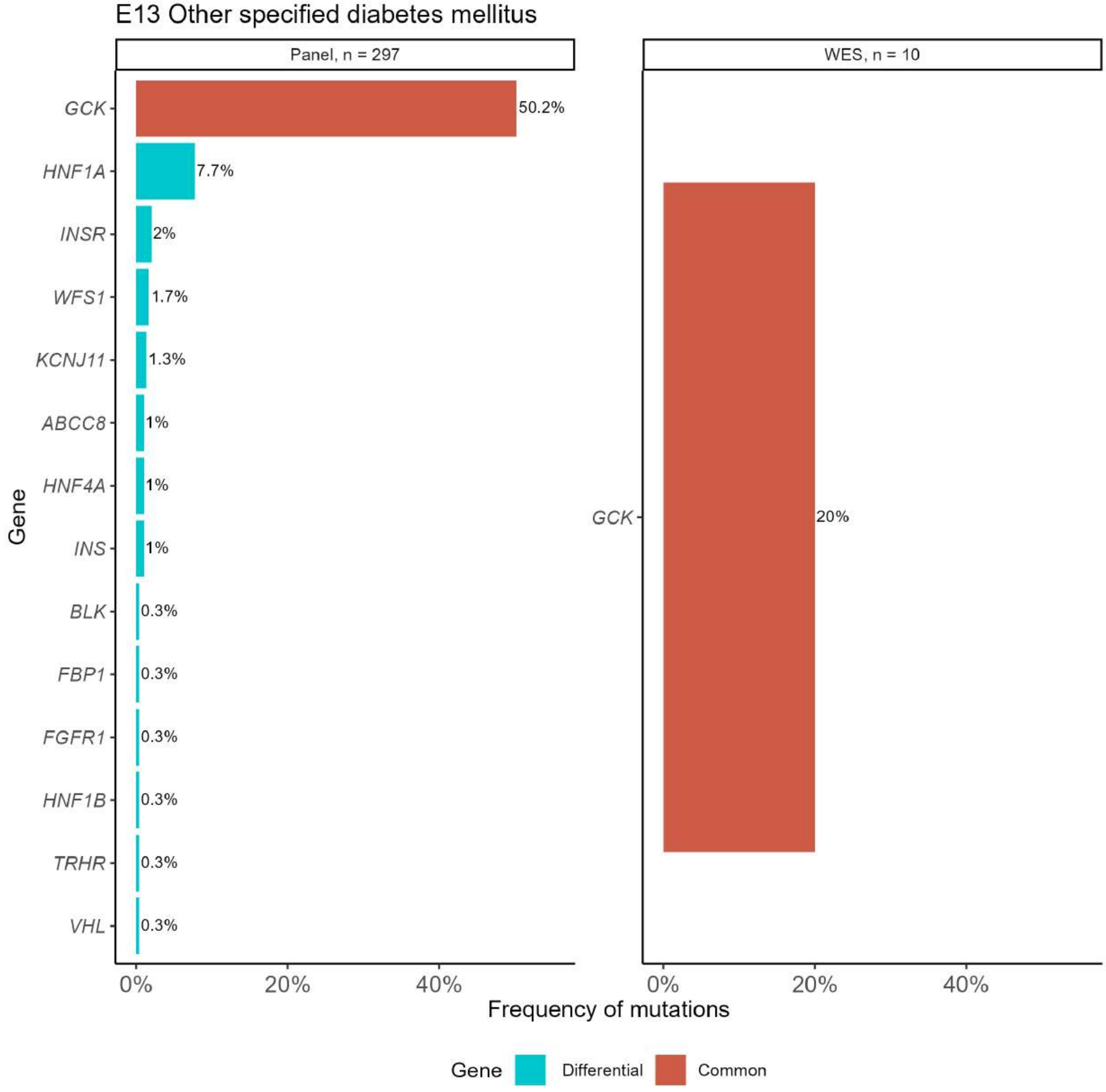
E13 Other specified diabetes mellitus
Panel, n = 297
WES, n = 10
GCK
HNF1A
INSR
WFS1
KCNJ11
ABCC8
HNF4A
INS
BLK
FBP1
FGFR1
HNF1B
TRHR
VHL
50.2%
7.7%
2%
1.7%
1.3%
1%
1%
1%
0.3%
0.3%
0.3%
0.3%
0.3%
0.3%
GCK
20%
Gene
0%
20%
40%
0%
20%
40%
Frequency of mutations
Gene
Differential
Common

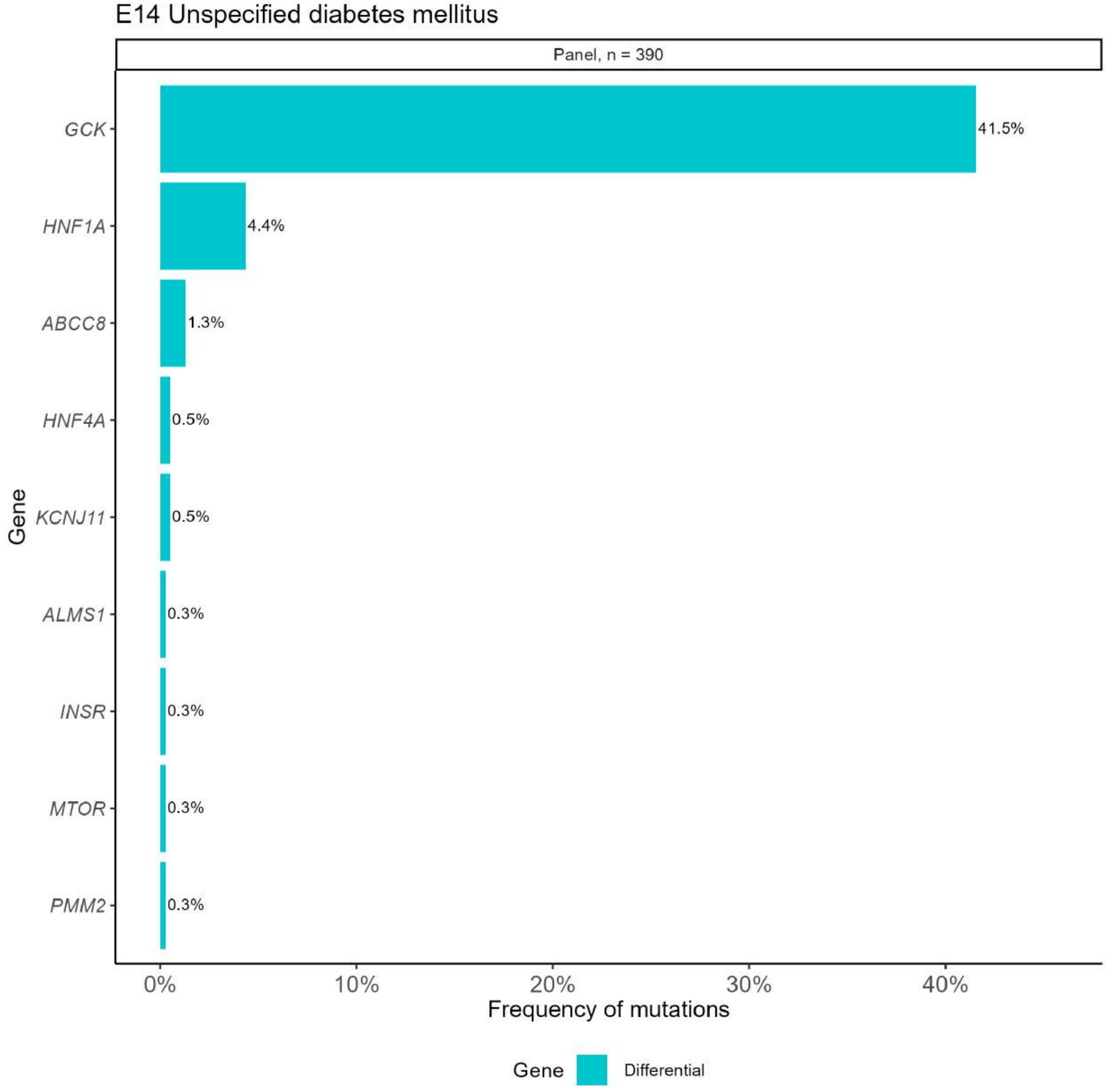

E14 Unspecified diabetes mellitus
Panel, n = 390
GCK
HNF1A
ABCC8
HNF4A
KCNJ11
ALMS1
INSR
MTOR
PMM2
Gene
41.5%
4.4%
1.3%
0.5%
0.5%
0.3%
0.3%
0.3%
0.3%
0%
10%
20%
30%
40%
Frequency of mutations
Gene
Differential

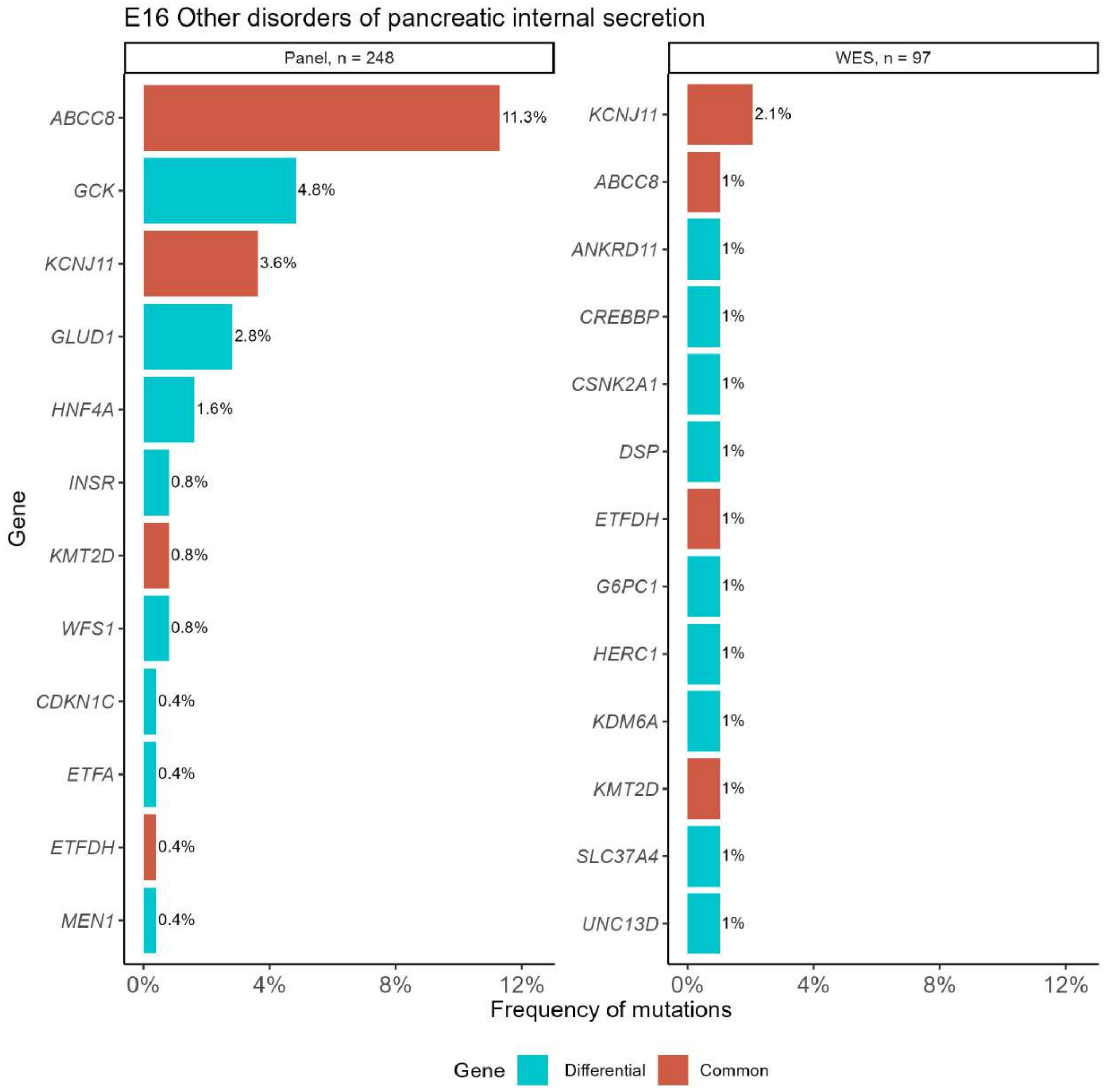
E16 Other disorders of pancreatic internal secretion
Panel, n = 248
WES, n = 97
ABCC8
GCK
KCNJ11
GLUD1
HNF4A
INSR
KMT2D
WFS1
CDKN1C
ETFA
ETFDH
MEN1
11.3%
4.8%
3.6%
2.8%
1.6%
0.8%
0.8%
0.8%
0.4%
0.4%
0.4%
0.4%
KCNJ11
ABCC8
ANKRD11
CREBBP
CSNK2A1
DSP
ETFDH
G6PC1
HERC1
KDM6A
KMT2D
SLC37A4
UNC13D
2.1%
1%
1%
1%
1%
1%
1%
1%
1%
1%
1%
1%
1%
Gene
0%
4%
8%
12%
Frequency of mutations
Gene
Differential
Common

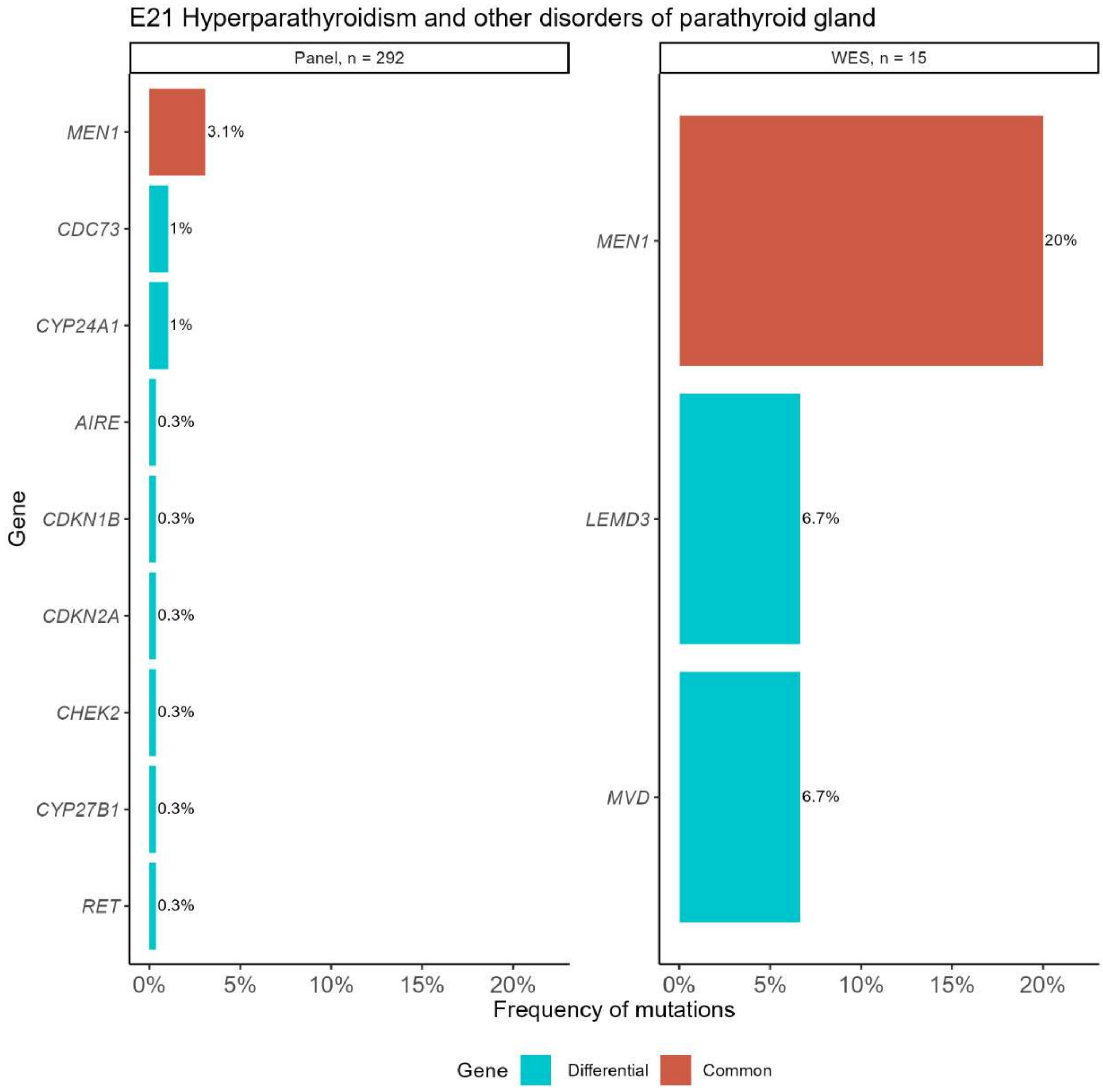

E21 Hyperparathyroidism and other disorders of parathyroid gland
Panel, n = 292
WES, n = 15
MEN1
CDC73
CYP24A1
AIRE
CDKN1B
CDKN2A
CHEK2
CYP27B1
RET
3.1%
1%
1%
0.3%
0.3%
0.3%
0.3%
0.3%
0.3%
MEN1
LEMD3
MVD
20%
6.7%
6.7%
Gene
0%
5%
10%
15%
20%
Frequency of mutations
Gene
Differential
Common

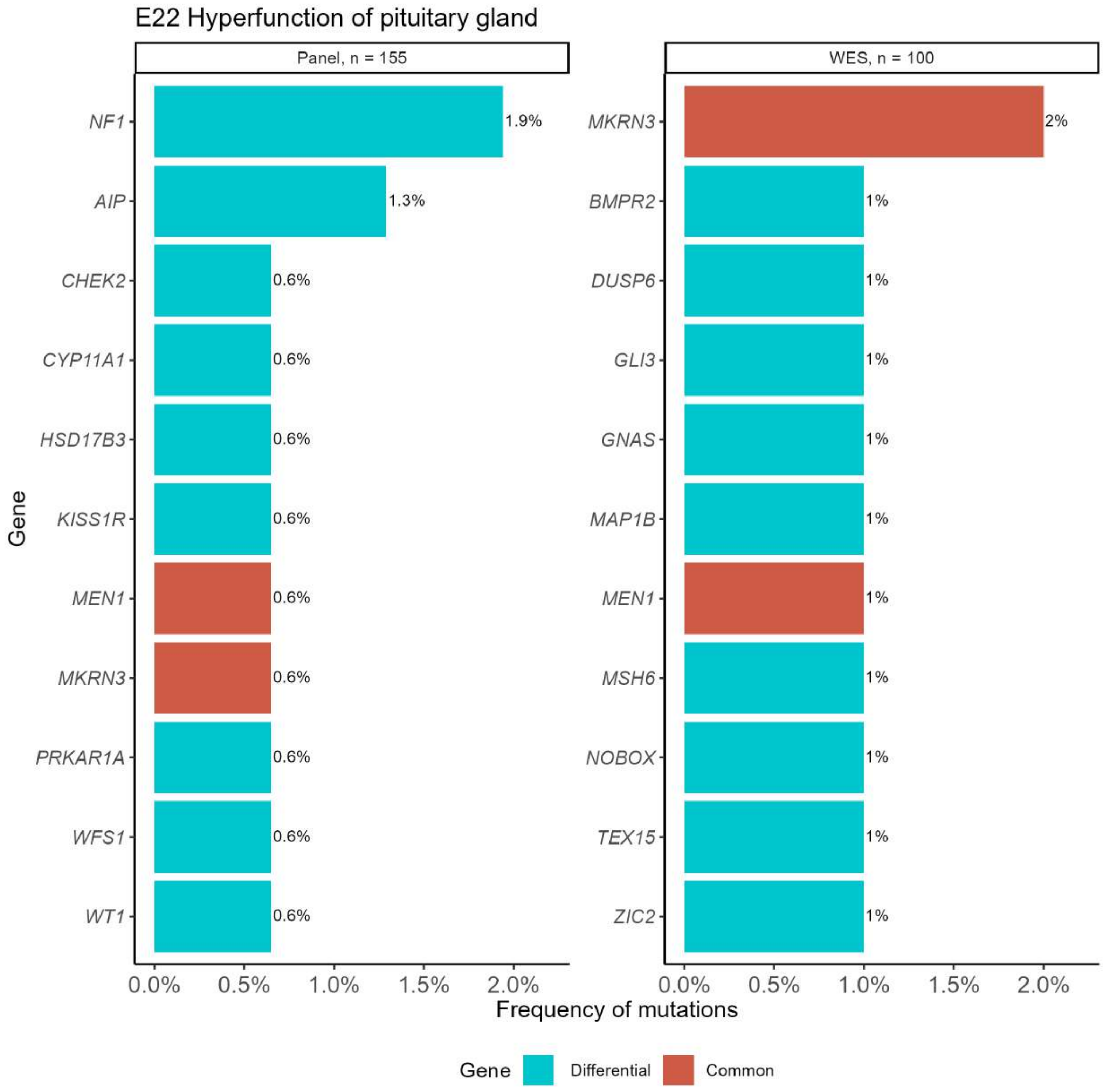

E22 Hyperfunction of pituitary gland
Panel, n = 155
WES, n = 100
NF1
AIP
CHEK2
CYP11A1
HSD17B3
KISS1R
MEN1
MKRN3
PRKAR1A
WFS1
WT1
1.9%
1.3%
0.6%
MKRN3
BMPR2
DUSP6
GLI3
GNAS
MAP1B
MEN1
MSH6
NOBOX
TEX15
ZIC2
2%
1%
Gene
0.0%
0.5%
1.0%
1.5%
2.0%
Frequency of mutations
Gene
Differential
Common

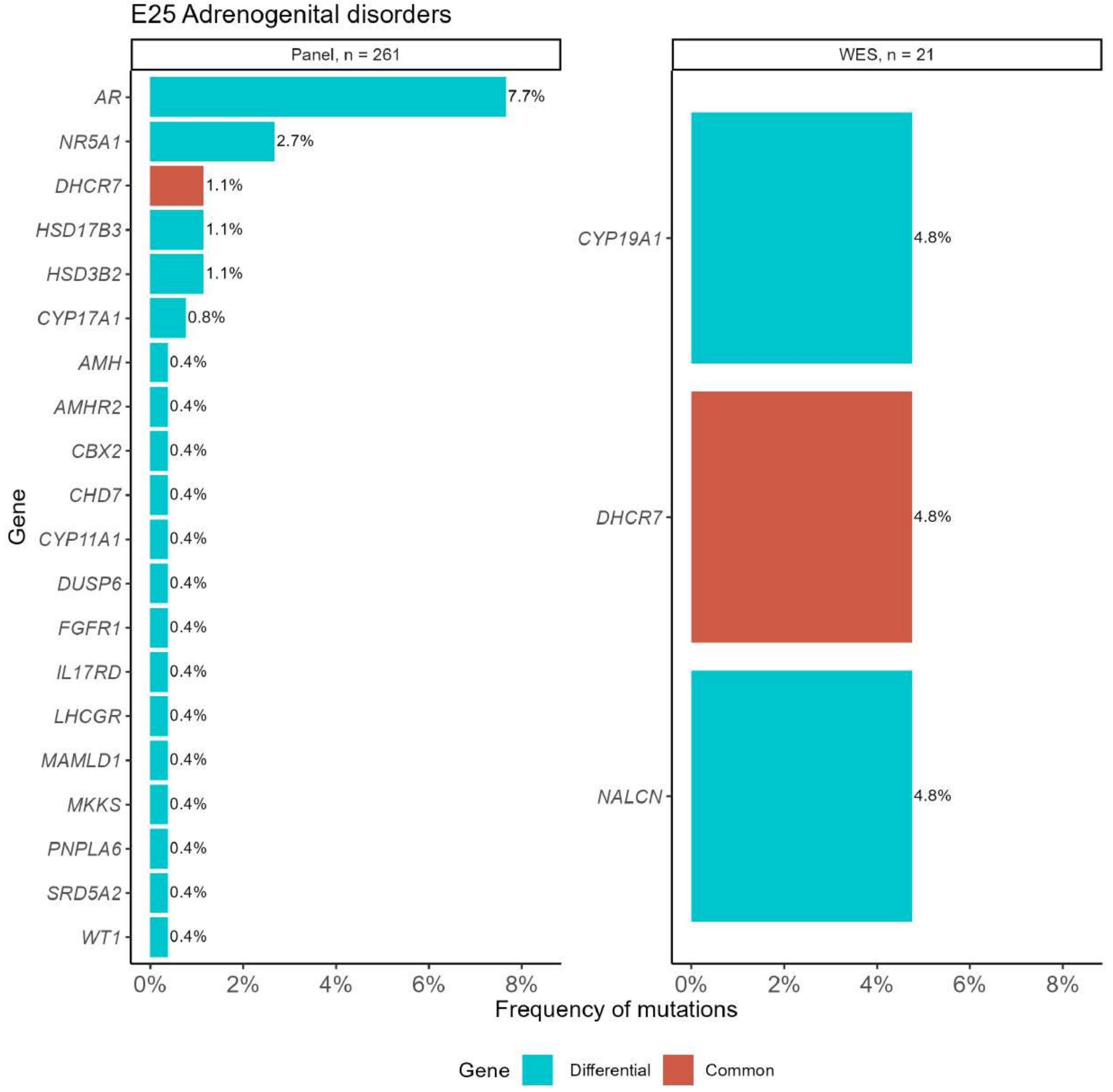
E25 Adrenogenital disorders
Panel, n = 261
WES, n = 21
Gene
AR
NR5A1
DHCR7
HSD17B3
HSD3B2
CYP17A1
AMH
AMHR2
CBX2
CHD7
CYP11A1
DUSP6
FGFR1
IL17RD
LHCGR
MAMLD1
MKKS
PNPLA6
SRD5A2
WT1
7.7%
2.7%
1.1%
1.1%
1.1%
0.8%
0.4%
0.4%
0.4%
0.4%
0.4%
0.4%
0.4%
0.4%
0.4%
0.4%
0.4%
0.4%
0.4%
0.4%
CYP19A1
DHCR7
NALCN
4.8%
4.8%
4.8%
0%
2%
4%
6%
8%
Frequency of mutations
Gene
Differential
Common

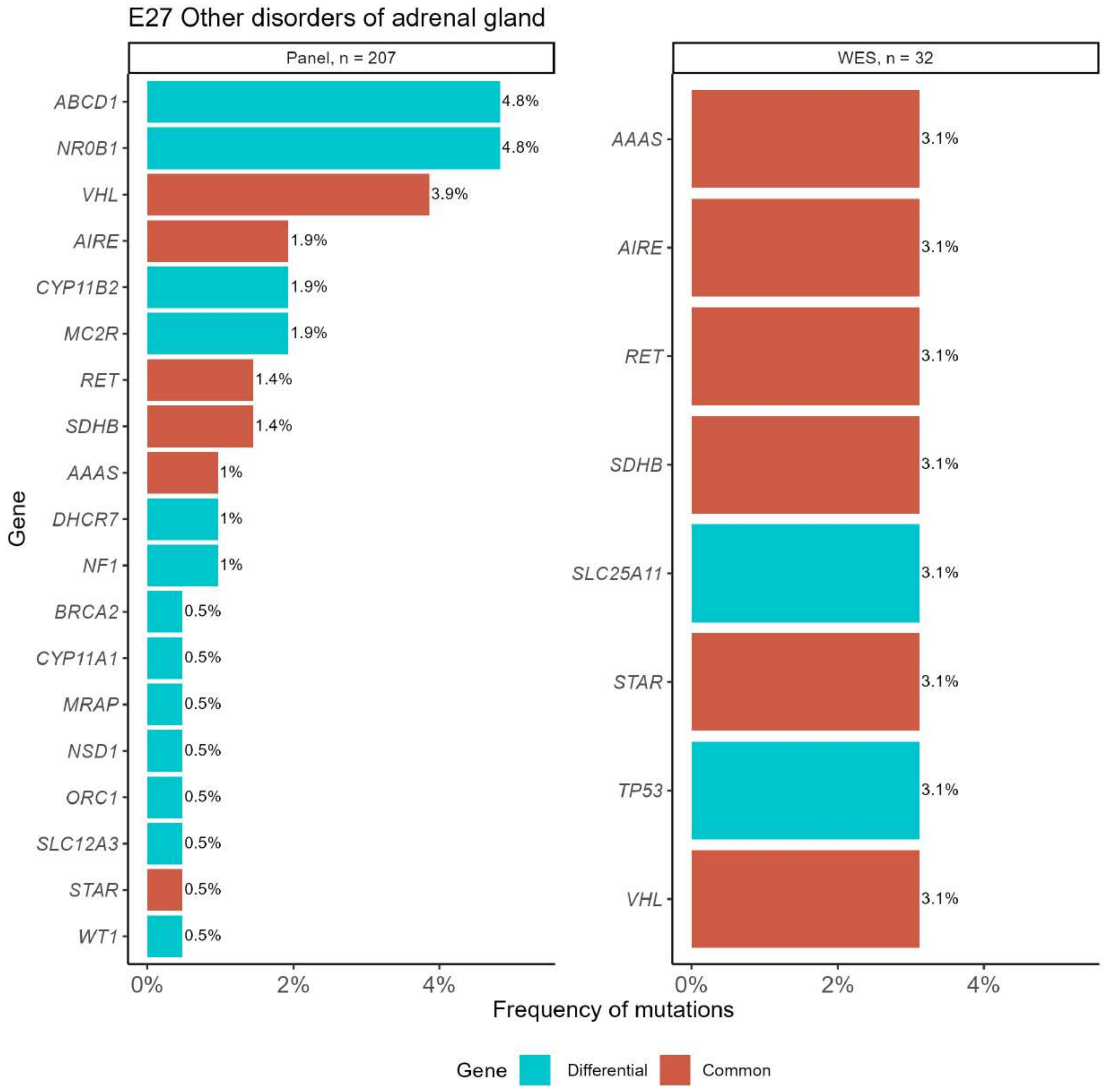

E27 Other disorders of adrenal gland
Panel, n = 207
WES, n = 32
ABCD1 4.8%
NR0B1 4.8%
VHL 3.9%
AIRE 1.9%
CYP11B2 1.9%
MC2R 1.9%
RET 1.4%
SDHB 1.4%
AAAS 1%
DHCR7 1%
NF1 1%
BRCA2 0.5%
CYP11A1 0.5%
MRAP 0.5%
NSD1 0.5%
ORC1 0.5%
SLC12A3 0.5%
STAR 0.5%
WT1 0.5%
AAAS 3.1%
AIRE 3.1%
RET 3.1%
SDHB 3.1%
SLC25A11 3.1%
STAR 3.1%
TP53 3.1%
VHL 3.1%
Gene
0%
2%
4%
Frequency of mutations
Gene
Differential
Common

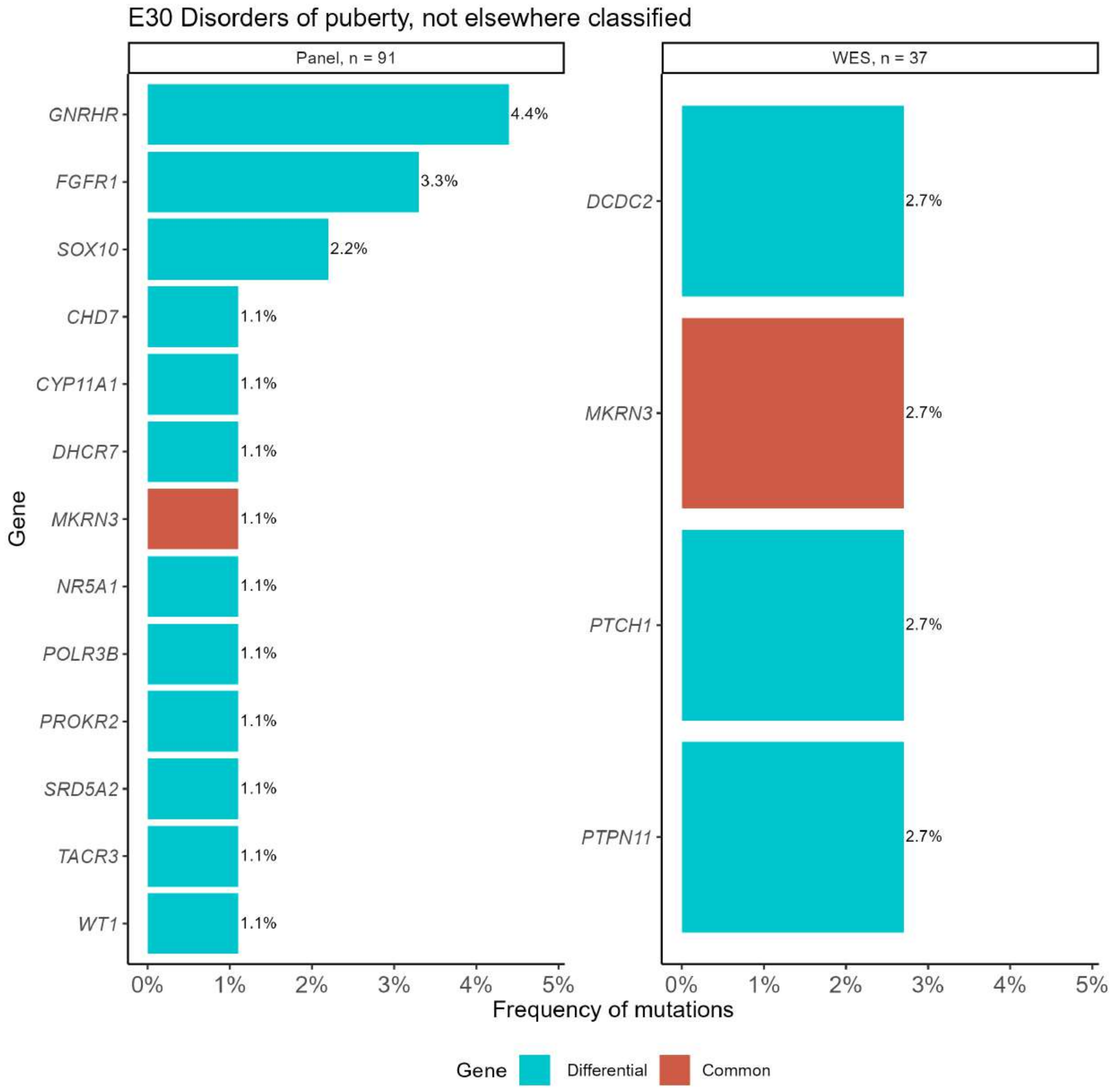

E30 Disorders of puberty, not elsewhere classified
Panel, n = 91
WES, n = 37
GNRHR
FGFR1
SOX10
CHD7
CYP11A1
DHCR7
MKRN3
NR5A1
POLR3B
PROKR2
SRD5A2
TACR3
WT1
4.4%
3.3%
2.2%
1.1%
1.1%
1.1%
1.1%
1.1%
1.1%
1.1%
1.1%
1.1%
1.1%
DCDC2
MKRN3
PTCH1
PTPN11
2.7%
2.7%
2.7%
2.7%
Gene
0%
1%
2%
3%
4%
5%
Frequency of mutations
Gene
Differential
Common

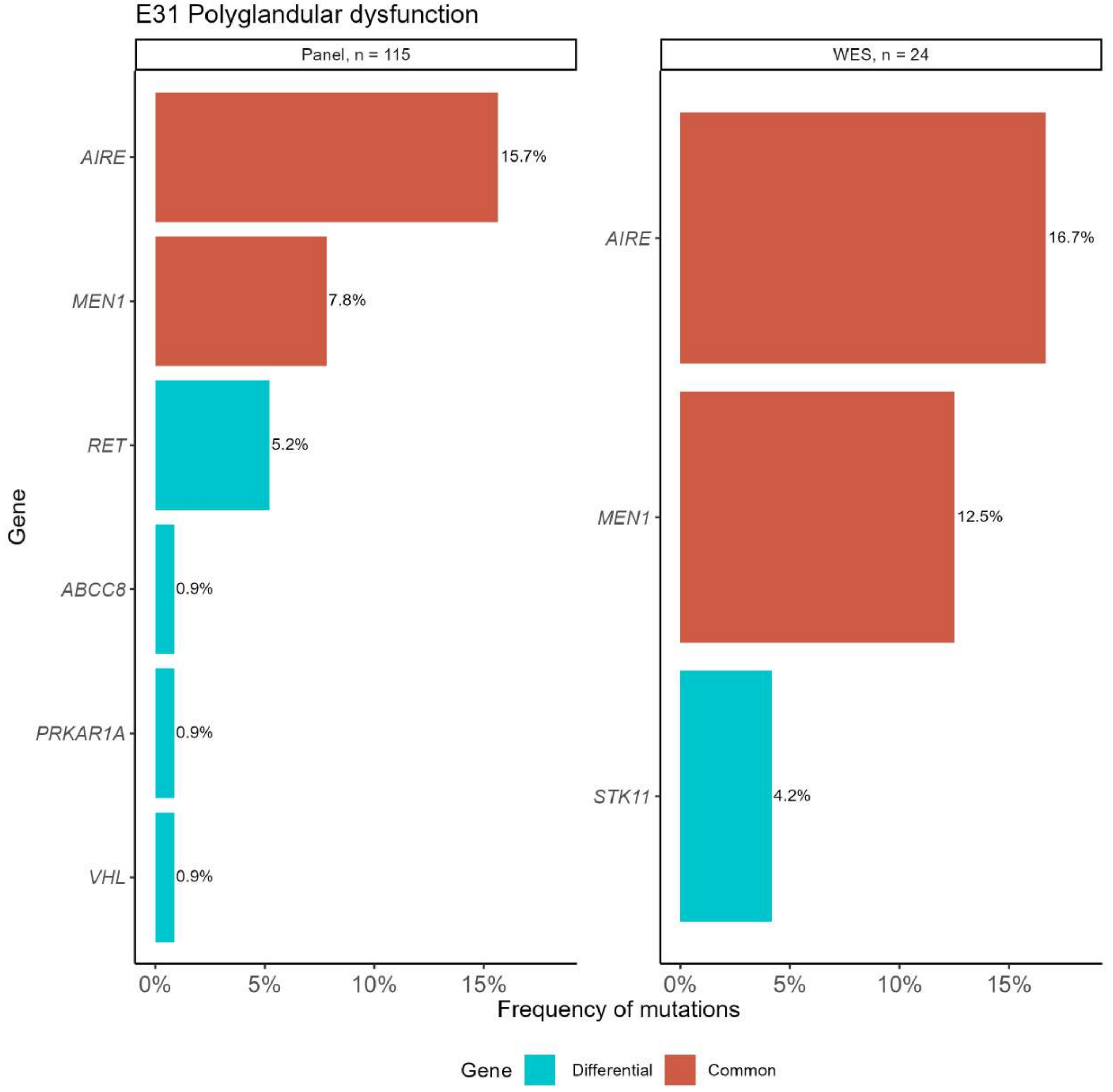
E31 Polyglandular dysfunction
Panel, n = 115
WES, n = 24
AIRE
MEN1
RET
ABCC8
PRKAR1A
VHL
STK11
15.7%
7.8%
5.2%
0.9%
0.9%
0.9%
16.7%
12.5%
4.2%
0%
5%
10%
15%
Gene
Frequency of mutations
Gene
Differential
Common

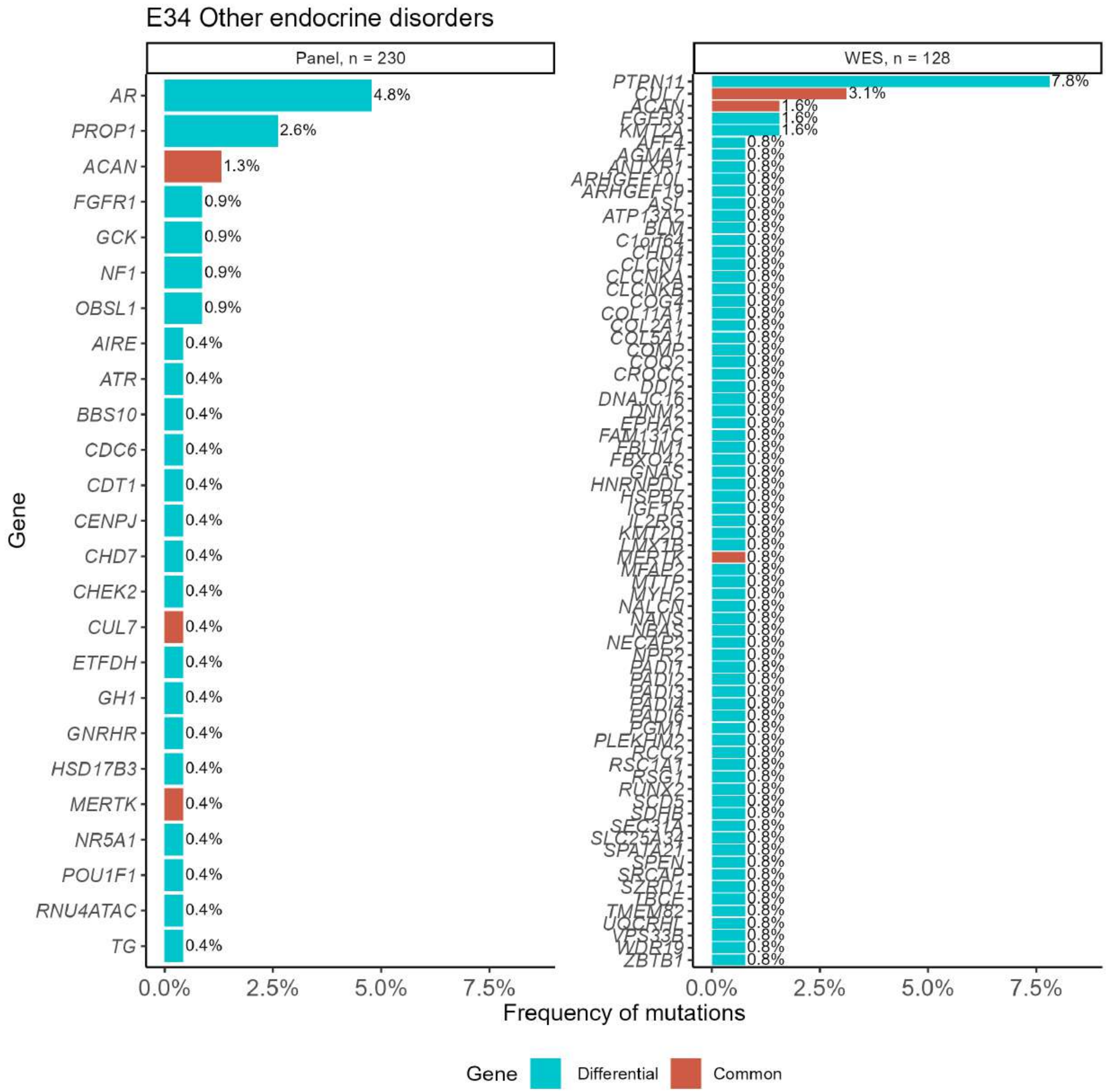
E34 Other endocrine disorders
Panel, n = 230
WES, n = 128
AR 4.8%
PROP1 2.6%
ACAN 1.3%
FGFR1 0.9%
GCK 0.9%
NF1 0.9%
OBSL1 0.9%
AIRE 0.4%
ATR 0.4%
BBS10 0.4%
CDC6 0.4%
CDT1 0.4%
CENPJ 0.4%
CHD7 0.4%
CHEK2 0.4%
CUL7 0.4%
ETFDH 0.4%
GH1 0.4%
GNRHR 0.4%
HSD17B3 0.4%
MERTK 0.4%
NR5A1 0.4%
POU1F1 0.4%
RNU4ATAC 0.4%
TG 0.4%
PTPN11 7.8%
CUL7 3.1%
ACAN 1.6%
FGFR3 1.6%
KMT2A 1.6%
0.8%
Gene
Frequency of mutations
0.0%
2.5%
5.0%
7.5%
Gene
Differential
Common

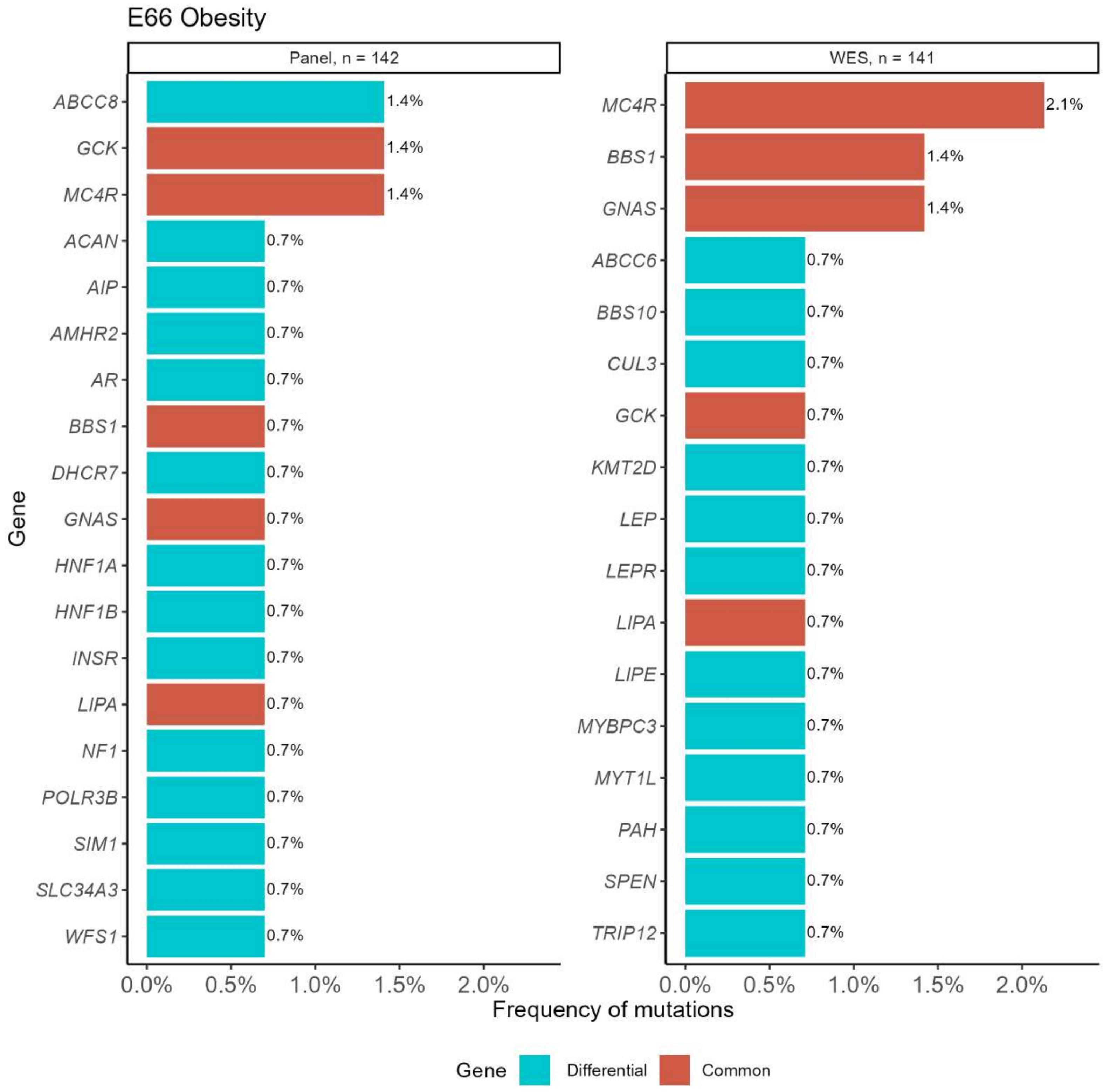
E66 Obesity
Panel, n = 142
WES, n = 141
ABCC8
GCK
MC4R
ACAN
AIP
AMHR2
AR
BBS1
DHCR7
GNAS
HNF1A
HNF1B
INSR
LIPA
NF1
POLR3B
SIM1
SLC34A3
WFS1
1.4%
1.4%
1.4%
0.7%
MC4R
BBS1
GNAS
ABCC6
BBS10
CUL3
GCK
KMT2D
LEP
LEPR
LIPA
LIPE
MYBPC3
MYT1L
PAH
SPEN
TRIP12
2.1%
1.4%
1.4%
0.7%
Gene
0.0%
0.5%
1.0%
1.5%
2.0%
Frequency of mutations
Gene
Differential
Common

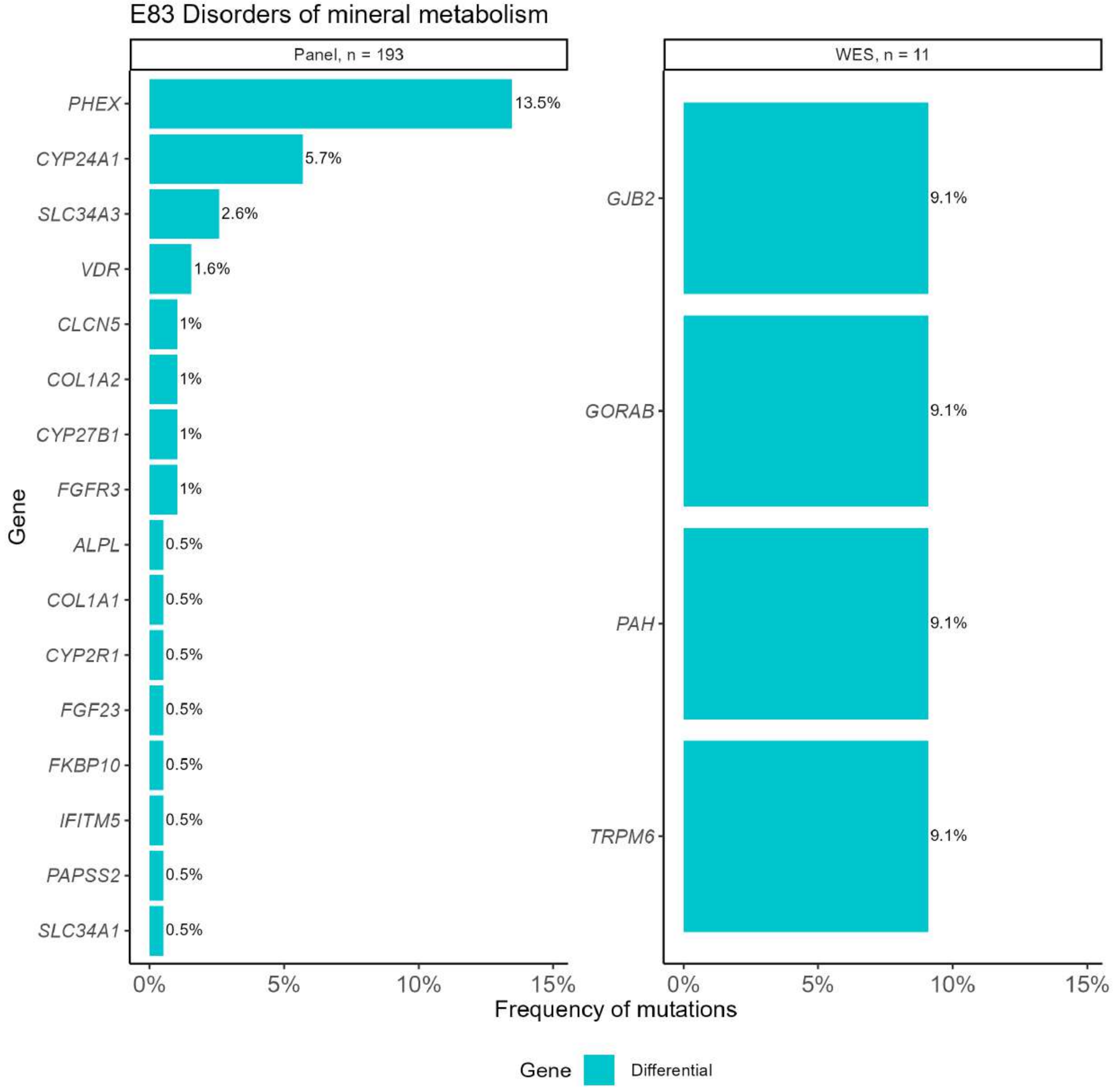

E83 Disorders of mineral metabolism
Panel, n = 193
WES, n = 11
PHEX
CYP24A1
SLC34A3
VDR
CLCN5
COL1A2
CYP27B1
FGFR3
ALPL
COL1A1
CYP2R1
FGF23
FKBP10
IFITM5
PAPSS2
SLC34A1
13.5%
5.7%
2.6%
1.6%
1%
1%
1%
1%
0.5%
0.5%
0.5%
0.5%
0.5%
0.5%
0.5%
0.5%
GJB2
GORAB
PAH
TRPM6
9.1%
9.1%
9.1%
9.1%
Gene
0%
5%
10%
15%
Frequency of mutations
Gene
Differential
