# Supplementary figures and images for: EndoGene database: reported genetic variants for 5,926 Russian patients diagnosed with endocrine disorders
Source: Front Endocrinol (Lausanne). 2025 Feb 18;16:1472754. doi: 10.3389/fendo.2025.1472754 (PMC11876052; doi:10.3389/fendo.2025.1472754)

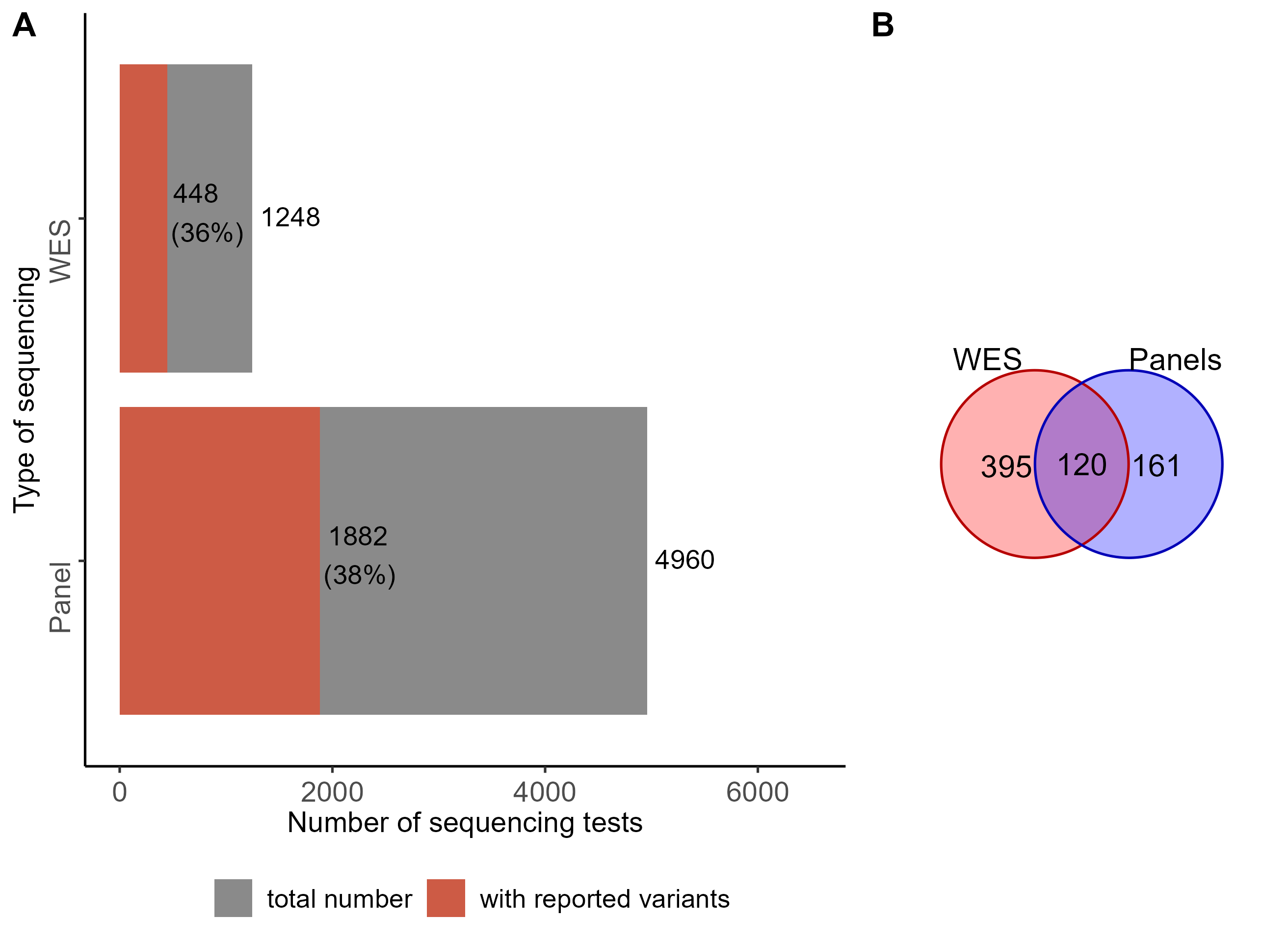

Supplement: Supplementary Figure 1 — The number of genes hosting genetic variants classified as being of pathogenic, likely pathogenic, and uncertain significance in the results of WES and panel NGS. [file Image1.png]
